# Supplementary material for: Basic interactions responsible for thymus function explain the convoluted medulla shape
Source: Proc Natl Acad Sci U S A. 2025 Jun 20;122(25):e2415288122. doi: 10.1073/pnas.2415288122 (PMC12207460; doi:10.1073/pnas.2415288122)
Supplement: Supplementary file 1 — Appendix 01 (PDF) [file pnas.2415288122.sapp.pdf]

Supporting Information for

Basic interactions responsible for thymic function explain convoluted medulla shape

David Muramatsu*, Henrik Weyer*, Florian Gartner, Erwin Frey

Corresponding author: Erwin Frey.
E-mail: frey@lmu.de

***These authors contributed equally to this work.**

This PDF file includes:

Supporting text
Figs. S1 to S7
Tables S1 to S2
SI References

Supporting Information Text

1. Experimental basis for the model and parameter choice

In the main text, we proposed a coarse-grained model of the most important interactions of thymic cross-talk that take part in medulla formation. In this section, we expand on the experimental evidence of the interactions and model parameters outlined in the main text. First, we discuss the simplifications of the known interactions and constituents of the thymus, focusing on the interactions taking place within the medulla in Sec. A. Subsequently, in Sec. B, we explain the parameter choices used in the simulations based on the experimental literature. Finally, in Sec. C, we derive the sensitivity function that modulates the chemotaxis in dependence of chemokine densities in the medulla, following an argument by Stevens and Othmer (1).

A. Model Abstractions.

A.1. Chemokines. Both cTECs and mTECs, as well as dendritic cells, emit chemokines that steer thymocytes in various stages into their appropriate microenvironments (for an overview of these interactions, see (2, 3)). In this publication, we mainly model interactions between autoreactive SP thymocytes and medullary tissue. Thus, we do not expect the production of chemokines from cTECs which act on earlier development stadia of thymocytes to play a role in these processes. Rather, these effects are subsumed in the effective, cortex-restricted production term of thymocytes (the term $p\phi$ in Eq. (1) in the main text).

SP and/or post-positive selection DP thymocytes are attracted to mTEC-produced chemokines CCL21 (4), CCL19 (5), CCL1 (6), CCL8 (6), EBI2-ligand 7α25-OHC (7), as well as the mTEC- and DC-produced chemokines CCL17 and CCL22 (8–10). While many of these chemokines aid in recruiting thymocytes to the medulla (6, 7, 9, 11–15), only CCL21 has so far been shown to significantly impact medullary morphology and size (6–9, 11, 12, 16, 17). At the same time, DCs are attracted to the medulla via diverse chemokines, see e.g. (18–21), with the DC subsets that express the highest levels of CCL17 and CCL22 also expressing CCR7, the chemokine receptor for CCL21 (10) and cDCs, and the most numerous DC subset being mainly located in the medulla (18, 22, 23).

Thus, as all these attractive chemokine gradients predominantly originate in the medulla, we conflate them into one generalized chemokine gradient.

A.2. mTECs. At a coarse level, mTECs can be divided into the mTEC^{lo} and mTEC^{hi} populations which express the two surface markers MHC-II and CD80 at low and high levels, respectively (24). These two populations can be split up into further phenotypes (10, 25–29) whose differentiation and proliferation is controlled by interaction with thymocytes (30–33).

These cell populations are functionally distinct and recent evidence points to two branches of differentiation within the mTEC population originating from the same progenitor (29). Within the proposed model, one branch differentiates into an Aire$^+$ cell line (29). As Aire$^+$ cells as a whole are presenting nearly all of the bodies protein (26, 34), this branch is tasked with inducing self-tolerance (35). Aire$^+$ cells are known to be denser at the CMJ, particularly in young mice, and this preference is attenuated in adult mice (32). The effect of this distribution on thymocyte localization, as well as the mechanism leading to this distribution of Aire$^+$ cells is a fascinating extant question that we do not cover here.

The other branch is marked by a high expression of CCL21 and Cytokeratin 5 (Krt5) (29). While a type of cell expressing high amounts of CCL21 has been identified to be enriched at the CMJ (36), Krt5 is used as a medullary marker (37), with Krt5$^+$ cells evenly distributed in the medulla, and only known to be abnormally distributed in clusters in *Fezf2*$^{-/-}$ mice (38). Thus, the production of the chemokine by a single mTEC density nearly homogeneous within the medullary region in the model appears reasonable.

A.3. Antigen presenting cells. Apart from mTECs, several discrete cell subsets are known to present antigen to thymocytes for negative selection in the medulla. While mTECs, DCs and B-Cells (34, 39) clonally delete thymocytes, only mTECs are known to induce a stimulatory capacity for the mTEC-proliferation signal in thymocytes (31). As we model activated thymocytes to possess stimulatory capacity for mTEC proliferation, only mTEC-activated thymocytes fall into this subset, while B cell and DC-activated thymocytes do not.

The fraction of DC-stimulated cells is estimated to make up between 30 and 70% of negatively selected thymocytes (40–44). For simplicity, in this publication, we assume the value to be 50%. As the vast majority of negative selection events in the medulla are split between DCs and mTECs (41) in two-photon microscopy experiments, we neglect the effect of B cells and only consider mTECs and DCs.

DC-activation of thymocytes (and the minor contribution due to B cells) can be seen as an effective degradation term of thymocytes that leads to an effective reduction of the production rate of thymocytes, see also Sec. B.3, B.6. Specifically, because DCs mostly localize to the medulla, see e.g. Ref. (18, 45), we approximate their density ρ_{DC} as being proportional to the mTEC density, i.e., one has $\rho_{\mathrm{DC}} \sim \rho_{\mathrm{M}}$. Therefore, thymocyte deletion by DCs can be modeled by $-k_{\mathrm{DC}}\rho_{\mathrm{T}}\rho_{\mathrm{DC}} \approx -\tilde{k}_{\mathrm{DC}}\rho_{\mathrm{T}}\rho_{\mathrm{M}}$. Given the rate of thymocyte recognition by mTECs $\bar{k}_{\mathrm{r}}$ and that mTEC recognition accounts roughly for one half of negatively selected thymocytes, one has $\bar{k}_{\mathrm{r}} \approx \tilde{k}_{\mathrm{DC}}$. Because only mTEC-activated thymocytes stimulate mTEC growth, only these thymocytes are converted into the activated model species ρ_{T}^*. As a result, the model equations for the thymocytes that include DC-mediated negative selection, can be approximated as

$$\partial_t\rho_{\mathrm{T}} = D_{\mathrm{T}}\nabla^2\rho_{\mathrm{T}} - \nabla(\rho_{\mathrm{T}}\mathbf{v}_{\mathrm{C}}) - 2\bar{k}_{\mathrm{r}}\rho_{\mathrm{T}}\rho_{\mathrm{M}} + \bar{p}\phi(\rho_{\mathrm{M}})\,, \tag{1}$$

$$\partial_t\rho_{\mathrm{T}}^* = D_{\mathrm{T}}\nabla^2\rho_{\mathrm{T}}^* - \nabla(\rho_{\mathrm{T}}^*\mathbf{v}_{\mathrm{C}}) + \bar{k}_{\mathrm{r}}\rho_{\mathrm{T}}\rho_{\mathrm{M}} - d\,\rho_{\mathrm{T}}^*\,. \tag{2}$$

Here, the thymocytes are produced with an average cortical production rate $\bar{p}$. Rescacaling the thymocyte density as $\tilde{\rho}_{\mathrm{T}} = \frac{\rho_{\mathrm{T}}}{2}$ leads to an effectively halved production rate, and a doubled conversion rate $2\bar{k}_{\mathrm{r}}\rho_{\mathrm{M}}$ which is the same for autoreactive and activated thymocytes:

$$\partial_t \tilde{\rho}_{\mathrm{T}} = D_{\mathrm{T}} \nabla^2 \tilde{\rho}_{\mathrm{T}} - \nabla(\tilde{\rho}_{\mathrm{T}} \mathbf{v}_{\mathrm{C}}) - 2\bar{k}_{\mathrm{r}} \tilde{\rho}_{\mathrm{T}} \rho_{\mathrm{M}} + \frac{\bar{p}}{2} \phi(\rho_{\mathrm{M}}) \,, \qquad [3]$$

$$\partial_t \rho^*_{\mathrm{T}} = D_{\mathrm{T}} \nabla^2 \rho^*_{\mathrm{T}} - \nabla(\rho^*_{\mathrm{T}} \mathbf{v}_{\mathrm{C}}) + 2\bar{k}_{\mathrm{r}} \tilde{\rho}_{\mathrm{T}} \rho_{\mathrm{M}} - d\, \rho^*_{\mathrm{T}} \,. \qquad [4]$$

Thus, re-writing $\tilde{\rho}_{\mathrm{T}} \to \rho_{\mathrm{T}}$, one arrives at Eq. (1, 2) of the main text with $k_{\mathrm{r}} = 2\bar{k}_{\mathrm{r}}$ and $p = \frac{\bar{p}}{2}$.

A.4. Thymocytes. T cells developing in the thymus consist of several subsets, out of which the $\alpha\beta - \mathrm{CD4}^+$ and $\alpha\beta - \mathrm{CD8}^+$ single positive (SP) subsets are the most abundant (39). Out of these, CD4 SP thymocytes possess the dominant stimulatory capacity for mTEC growth (31, 32), with other subsets playing a subordinate role (46). In this publication, we mainly focus on the functional role of thymocytes for mTEC differentiation, and thus, model all thymocytes that induce mTEC proliferation upon antigen recognition as the same effective cell species.

When considering the spatial distribution of thymocytes with respect to their environment, we introduce a population of tolerant thymocytes, as they are not degraded due to negative selection, and thus have different kinetics. For simplicity, we use the parameters of CD4 SP tolerant thymocytes for this population. However, all tolerant thymocytes have the same underlying kinetics of production in the cortex, transition to the medulla, and eventual exit out of the thymus, and thus share the same phenomenology.

CD4 SP thymocytes that are self-reactive also include the subset of regulatory T cells ($\mathrm{T_{reg}}$). Since, to our knowledge, there exists no data on the stimulatory capacity of $\mathrm{T_{reg}}$ cells on mTEC proliferation and differentiation, we do not explicitly model this subset. However, $\mathrm{T_{reg}}$ cells are known to preferentially arise in response to recognizing sparsely presented antigen (47), such that their kinetics may be different from general negatively selected CD4 SP thymocytes. Thus, if $\mathrm{T_{reg}}$ cells do possess stimulatory capacity in thymic cross-talk, an additional thymocyte population with stimulatory capacity might need to be introduced, potentially yielding interesting new phenomenology regarding the respective cell density distributions.

B. Parameters. The model parameters used for the results displayed in the main text are based on experimental findings, as we detail below. Taken together, the rates for the simulation reflecting the wild-type conditions can be found in Table I of the main text and for all other simulations in Tables S1, S2 of the SI.

B.1. Medullary and cortical volume. The volume of one thymic lobe has been estimated at 25 - 40mm^3 (48, 49), whereas the medullary volume have been measured to be around 10% (2.5mm^3) (48) and computed by us to be 20% (10mm^3) from the cortex and medulla volumes measured in (49). Thus, the cortical volume ranges between 90% (23mm^3) (48) and 80% (30mm^3) (49). Although the thymic volume itself is not a parameter in the analysis performed in this publication, it is necessary to estimate the rate of autoreactive thymocyte production.

B.2. Medullary length scales. We estimate the typical length scale (half wavelength, see Sec. B.2) of the cortex-medulla pattern to be around 1mm, and the width of the medullary branches to be around 500µm, based on histological sections in several studies (16, 17, 48, 50).

B.3. Production of autoreactive thymocytes. The production rate of autoreactive thymocytes has not been quantified. We therefore estimate it from measured selection rates by assuming a steady-state turnover: The rates for the selection of autoreactive thymocytes in the DP and SP stadium have been estimated experimentally in Refs. (51, 52), in good accordance with the fraction of autoreactive DP and SP thymocytes that undergo negative selection measured by Ref. (40). Since in our model, the inflow of autoreactive thymocytes is equal to the outflow by negative selection in steady state, the average rate of selection of one thymocyte differentiation stage must be equal to the average rate of production of thymocytes that are selected in this stadium.

Commonly, only CD4 SP thymocytes are thought to impact medullary growth, see e.g. Refs. (31, 40, 52). Thus, we consider only this thymocyte population to stimulate mTEC growth in our model. Stritesky et al. (52) found the total rate of negative selection — and thus the total production if we assume a steady-state turnover of the thymocytes — of autoreactive CD4 SP thymocytes to be $p_{\mathrm{tot}} = 1.66 \cdot 10^5 \mathrm{h}^{-1}$. With a cortical volume $V_{\mathrm{C}} = 23 - 30\mathrm{mm}^3$, the lower bound for the production rate of self-reactive thymocytes is

$$\bar{p} = \frac{p_{\mathrm{tot}}}{V_{\mathrm{C}}} = \frac{1.66 \cdot 10^5}{30 \cdot 10^9} \mathrm{h}^{-1}\, \mu\mathrm{m}^{-3} \approx 5 \cdot 10^{-6} \mathrm{h}^{-1}\, \mu\mathrm{m}^{-3} \,. \qquad [5]$$

As discussed in Sec. A.3, we estimate the total number of mTEC-stimulating activated thymocytes to be half that of total activated CD4 SP thymocytes, as the other half is activated by DCs. Thus, the production rate of thymocytes that undergo selection by mTECs, is

$$p = \frac{\bar{p}}{2} = 2.5 \cdot 10^{-6} \mathrm{h}^{-1}\, \mu\mathrm{m}^{-3} \,. \qquad [6]$$

We note that post-positive selection DP thymcoytes can localize into the medulla (9, 15). However, to our knowledge, it is not conclusively known whether these cells can drive mTEC differentiation. Thus, depending on the extent of both localization and differentiation signaling by post-positive selection DP thymocytes, the thymocyte production rate may have to be corrected to a larger value.

B.4. Thymocyte production in the cortex. DP thymocytes are located in the cortex, where they receive signals for positive selection (2). After receiving these signals, they differentiate and transition into the medulla (9, 11, 13–15). To reflect this dynamic in our model, we localize SP thymocyte production to the cortex using the function

$$\phi(\mathbf{x}) = \frac{1}{1 + (\rho_M(\mathbf{x})/\mu)^2}\,, \qquad [7]$$

with the parameter μ chosen such that the function falls off from one to zero around $\rho_{\mathrm{M}} \sim \mu$. We discuss the choice of μ in Sec. B.13.

B.5. Production rate of tolerant CD4 SP thymocytes. CD4 SP thymocytes survive negative selection at approximately half the rate with which they are negatively selected (52). However, since we are only considering half of the negatively selected thymocytes in their production rate p, we approximate the production rate of tolerant thymocytes as $p_{\mathrm{T}}^0 = p$.

B.6. Antigen recognition. In the thymus medulla, antigen is presented at different frequencies, with some antigens being expressed ubiquitously, and others, so-called *tissue restricted antigens*, are expressed sparsely (34). The timing and extent of the negative selection against ubiquitous (OVAp) (53) and sparse antigen (RIPmOVA) (54) have been analyzed in overlay experiments, where specific thymocytes are overlaid onto thymic slices containing their cognate antigen. In the case of ubiquitous antigen, the activation of thymocytes is almost immediate, with a two–hour lag-period, after which the phagocytosis by randomly encountered phagocytes sets in (53). In the experiment with sparse antigen selection, the lag extends beyond three hours, but in both experiments, deletion happens within the first nine hours, and there is little change in living cell numbers afterward (53, 54).

We choose our rates to be roughly in accordance with these results, with the mTEC-based activation rate at $\bar{k}_{\mathrm{r}} = \frac{1}{2}\mathrm{h}^{-1}$, and thus, the effective activation rate

$$k_{\mathrm{r}} = 2\,\bar{k}_{\mathrm{r}} = 1\,\mathrm{h}^{-1}\,, \qquad [8]$$

as detailed in Sec. A.3.

The validity of the RIPmOVA system as a model for sparse antigen presentation has been challenged (47, 55, 56), and lower rates of antigen presentation than the ones that are prevalent in this system would lead to lower rates of antigen recognition.

To estimate a lower bound for the thymic selection rate, we consider the fraction of autoreactive T cells in the entire body. While it has been suggested that around one-third of the body's T cell repertoire is autoreactive (47, 57, 58), it is likely that almost all thymocytes that are autoreactive against ubiquitous antigen and those which react strongly against sparse antigen are selected in the thymus (47, 57). Thus, we assume the time scale of selection against these thymocytes to be at least an order of magnitude faster than the time scale of thymocyte egress (around 4-5 days (59)), as otherwise, a large fraction of these autoreactive thymocytes would escape.

Nonetheless, it is conceivable that the rate of thymocyte selection is within the span of 1/1h to 1/5d, the rate of thymic egress. The fundamental mechanism underlying our model is not abolished by this parameter and a convoluted medulla forms due to thymocyte production and degradation on both ends of the rate's spectrum.

B.7. Decay rate of activated thymocytes. Upon antigen recognition, thymocytes upregulate phagocytosis-inducing factors (53), and thymocyte counts decrease sharply after the appearance of the first death markers, and from the data presented in (53), we estimate a half-life of activated thymocytes of one hour.

B.8. Exit rate of SP thymocytes. SP thymocytes exit the thymus with a rate of approximately five days (59), if they are not negatively selected. Thymocytes can exit the thymus after maturing from both cortex and medulla (11, 60). Thus, for simplicity we choose a uniform exit rate $k_{\mathrm{ex}} = (5\mathrm{d})^{-1} \approx 0.008\mathrm{h}^{-1}$.

B.9. mTEC death rate. The half-life of mTECs has been estimated between 0.9 w for young mice, and 6 - 11 w for adult mice (61–63), yielding a decay rate of $k_{\mathrm{m}} = 0.0004 - 0.005\mathrm{h}^{-1}$. Both ends of the thus given spectrum yield qualitatively the same morphology, as long as the ratio of mTEC proliferation rate k_{b} and mTEC death rate k_{m} are held constant. We choose the faster dynamics with a value of $k_{\mathrm{m}} = 0.005\mathrm{h}^{-1}$, representing the mTEC dynamics in young mice (63) as the patterning of the thymus will mostly happen during this stage. The high turnover of mTECs has been discussed in the context of the expression of the auto-immune regulator AIRE (62). Cells that express this protein have particularly high turnover rates and, at the same time, express a wide variety of antigen (35). As DCs are known to cross-present mTEC-derived antigen (64), high turnover of AIRE^+ mTECs may help the transfer of antigen to DCs (64).

B.10. Medullary mTEC density. The density of mTECs in the medulla has been measured at $2 - 3 \cdot 10^{-4} \mu\mathrm{m}^{-3}$ for 5wk and around $2.5 \cdot 10^{-4} \mu\mathrm{m}^{-3}$ for 12wk old mice by Ref. (65) and around $8 \cdot 10^{-4} \mu\mathrm{m}^{-1}$ by Ref. (66) at ten days after birth, the former of which are in good agreement with values by Ref. (49) who found a total number of mTECs of $\sim 2.5 \cdot 10^6$ cells and a thymic right lobe volume of approximately $10\mathrm{mm}^3$, 5wk after birth. We thus adopt this value of $\rho_{\mathrm{M}} = 2.5 \cdot 10^{-4} \mu\mathrm{m}^{-3}$ in our model parameters.

B.11. mTEC proliferation rate. The mTEC proliferation rate $k_{\mathrm{b}} = 3 \cdot 10^3 \mu\mathrm{m}^3\,\mathrm{h}^{-1}$ is chosen to reproduce a medullary mTEC density of $\rho_{\mathrm{M}} = 2.5 \cdot 10^{-4} \mu\mathrm{m}^{-3}$ in the wt steady state (see Sec. B.10), with a three-fold higher medullary density allowed by the carrying capacity.

B.12. mTEC carrying capacity. The carrying capacity K limits the production of new mTECs and thus sets the density above which mTECs do not proliferate. In early embryonic development, the mTEC densities are about three-fold higher (66) than at the stage of development in which the three-dimensional structure of the thymus was analyzed (48, 67), implying a considerable range of possible densities. We chose $K = 7.5 \cdot 10^{-4} \mu\text{m}^{-3}$ such that mTEC densities are capped but a three-fold increase in mTEC densities compared to the chosen wild-type density $2.5 \cdot 10^{-4}\mu\text{m}^{-3}$ is allowed.

B.13. mTEC density scale. As mentioned in Sec. B.10, we choose the mTEC density to be $\rho_\text{M} \approx 2.5 \cdot 10^{-4}\mu\text{m}^{-3}$ inside the medulla. Thus, we choose a value of $\mu = 10^{-4}\mu\text{m}^{-3}$ for the mTEC density scale.

B.14. Chemokine decay rate. The rate of chemokine decay due to proteases has been estimated between 0.72h^{-1} and 18h^{-1} by Ref. (68) based on previous studies (69, 70). However, since chemokines may be taken up by their cognate receptors and atypical chemokine receptors (71), this rate may be larger. Here, we use a rough estimate of $k_\text{e} = 10\text{h}^{-1}$. While ten-fold larger values impose narrower cortical regions, we checked that they do not qualitatively change the medullary morphology in the wild-type case.

B.15. Chemokine production rate. The average concentration of CCL21 has been measured to be $\sim$ 5ng per mg thymus by Ref. (72) and 2ng/mg by Ref. (73). At a mass of CCL21 of 14.5 kDa (74), this translates to densities of $160 - 400$nM. Using the chemokine decay rate, as well as the medullary mTEC densities described above, we choose our chemokine production rate to result in the approximate average density of about 200nM in the wild-type steady-state and arrive at a production rate of $1.25 \cdot 10^{-2}\text{M} \cdot \mu\text{m}^3\text{h}^{-1}$.

B.16. Chemokine Diffusivity. Chemokine diffusivity in serum has been estimated at $6.5 \cdot 10^{-7}\text{cm}^2\text{s}^-1 = 2.34 \cdot 10^5 \mu\text{m}^2\text{h}^{-1}$ in Ref. (68) based on experimental data from Ref. (75–77). Since the chemokine diffuses through the interstitium, we use a ten-fold lower value, however, choosing the original value does not change the morphology of the system for the chosen 'wt' parameters.

B.17. Thymocyte Diffusivity. The thymocyte motility has been measured to be approximately $D_\text{T} = 400\mu\text{m}^2\text{min}^{-1} = 24 \cdot 10^3 \mu\text{m}^2\text{h}^{-1}$ by Ref. (78), which we use in our numerical analysis.

Tolerant thymocytes that do not undergo chemotaxis are distributed approximately evenly in cortex and medulla in our model, even though they are produced in the cortex. This can be explained as follows: Tolerant thymocytes spend a time of $t_\text{med} = 4 - 5$d inside the thymus medulla (59), see Sec. B.8. As without chemotaxis, these cells perform random motion with the above given diffusivity, within that time they cover a distance of about $l_\text{diff} \sim \sqrt{t_\text{med} \cdot D_\text{T}} = \sqrt{5 \cdot 24\text{h} \cdot 24 \cdot 10^3 \mu\text{m}^2\text{h}^{-1}} \approx 1.7$mm, or about half the chosen simulated volume. Thus, a time of five days is enough for equilibrating the tolerant thymocyte density between the cortex, where they are produced, and the medulla.

B.18. Chemotaxis strength. The strength of chemotaxis T is a free parameter, since we model the combined effect of the chemokines, nor is it known for chemotaxis in the thymic environment. In the simulations, we choose a value of $10^5 \mu\text{m}^2\text{h}^{-1}$ which yields the desired patterns.

B.19. Chemokine binding affinity. The equilibrium constant of receptor-chemokine binding for CCL21-CCR7 has been estimated between 1.6 and 10nM (79–81), here we take the choice of 5nM, orienting ourselves on the estimate by Ref. (68), which we use as a generic chemokine binding constant.

C. Chemotaxis sensitivity function. Chemokine receptor binding kinetics have previously been successfully used to model CCR7-dependent T cell and DC chemotaxis (68, 82). We, also include this type of signaling as a chemokine density-dependent sensitivity function $\chi(\rho_\text{C})$ in the advection term of our model.

Therefore, we choose simple receptor binding kinetics (83) of the form

$$R + C \underset{k_\text{off}}{\overset{k_\text{on}}{\rightleftharpoons}} R \cdot C \,, \tag{9}$$

where R and C signify unbound receptor and chemokine molecules, and $R \cdot C$ signifies a bound receptor-chemokine complex, following the derivation by Stevens and Othmer (1). In steady state, binding and unbinding are balanced, which reads following mass-action kinetics

$$k_\text{on}[R][C] = k_\text{on}\left([R_\text{tot}] - [R \cdot C]\right)[C] = k_\text{off}[R \cdot C] \,, \tag{10}$$

such that

$$[R \cdot C] = \frac{k_\text{on}\,[R_\text{tot}]\,[C]}{k_\text{off} + k_\text{on}[C]} = [R_\text{tot}]\,\frac{[C]}{K_\text{OffOn} + [C]} \,, \tag{11}$$

where $K_\text{OffOn} = {}^{k_\text{off}}/_{k_\text{on}}$ is the dissociation constant of chemokine-receptor binding and $[R_\text{tot}]$ is the concentration of all bound and unbound receptors in the cell we consider. Since the cell senses its surrounding chemokine gradient via the gradient of bound receptors on its surface, we calculate

$$\begin{aligned} \nabla[R \cdot C] &= \nabla\,[R_\text{tot}]\,\frac{[C]}{K_\text{OffOn} + [C]} \\ &= [R_\text{tot}]\,\frac{K_\text{OffOn}}{\left(K_\text{OffOn} + [C]\right)^2}\nabla[C] \,, \end{aligned} \tag{12}$$

such that the total receptor density $[R_{\rm tot}]$ can be absorbed into the factor for chemotaxis strength, T, and the sensitivity function depends only on the chemokine density $[C] = \rho_C$,

$$\chi(\rho_C) = \frac{K_{\rm OffOn}}{(K_{\rm OffOn} + \rho_C)^2} \,. \qquad [13]$$

2. Linear Stability Analysis and model reduction

In this section, we present the mathematical analysis of the model equations. In the first subsection, we discuss the linear stability analysis (LSA) of the full model. The LSA allows one to determine the parameter region in which uniform density distributions of the cells and the chemoattractant (the homogeneous steady state, HSS) become unstable due to spatially inhomogenenous perturbations (lateral instability). In this regime, the system spontaneously segregates into cortical and medullary regions (cf. gray-shaded region in main text Fig. 4(A)).

In the following two subsections, we reduce the full model to a chemotaxis-free system and to a system without Turing instability. Using quasi–steady–state (QSS) approximations, we relate these systems to two-component Keller–Segel and Turing reaction–diffusion systems. This reduction shows explicitly that the two instability mechanisms of the full model, discussed in Sec. III.A in the main text, are the chemotaxis-driven instability conceptualized by the Keller–Segel model and the Turing reaction–diffusion instability.

A. Original model. In our spatial thymic cross-talk model, the densities of thymocytes, activated thymocytes, mTECs, and chemokines are given by the equations [cf. Eqs. (1-4) in the main text]

$$\partial_t \rho_{\rm T} = D_{\rm T}\nabla^2\rho_{\rm T} - \nabla(\rho_{\rm T} v_{\rm C}) - k_{\rm r}\rho_{\rm T}\rho_{\rm M} + p\,\phi(\rho_{\rm M})\,, \qquad [14a]$$

$$\partial_t \rho^*_{\rm T} = D_{\rm T}\nabla^2\rho^*_{\rm T} - \nabla(\rho^*_{\rm T} v_{\rm C}) + k_{\rm r}\rho_{\rm T}\rho_{\rm M} - d\,\rho^*_{\rm T}\,, \qquad [14b]$$

$$\partial_t \rho_{\rm M} = D_{\rm M}\nabla^2\rho_{\rm M} + k_{\rm b}\rho^*_{\rm T}\rho_{\rm M}\left(1 - \frac{\rho_{\rm M}}{K}\right) - k_{\rm m}\rho_{\rm M}\,, \qquad [14c]$$

$$\partial_t \rho_{\rm C} = D_{\rm C}\nabla^2\rho_{\rm C} + k_{\rm e}\rho_{\rm M} - k_{\rm c}\rho_{\rm C}\,, \qquad [14d]$$

where $\phi(\rho_{\rm M}) = (1 + (\rho_{\rm M}/\mu)^2)^{-1}$ and $\mathbf{v}_{\rm C} = T\chi(\rho_{\rm C})\nabla\rho_{\rm C}$ have been introduced in the main text. Defining the vector $\boldsymbol{\rho} = (\rho_{\rm T}, \rho^*_{\rm T}, \rho_{\rm M}, \rho_{\rm C})^T$, these equations can be written more compactly as

$$\partial_t \boldsymbol{\rho} = \nabla\left[\mathbf{M}(\boldsymbol{\rho})\nabla\boldsymbol{\rho}\right] + \mathbf{f}(\boldsymbol{\rho})\,. \qquad [15]$$

Here, the mobility matrix $\mathbf{M}$ accounts for the diffusion coefficients and chemotaxis terms and $\mathbf{f}$ contains the growth, conversion, and decay terms. Thus, they are given by

$$\mathbf{M}(\boldsymbol{\rho}) = \begin{pmatrix} D_{\rm T} & 0 & 0 & -T\chi(\rho_{\rm C})\rho_{\rm T} \\ 0 & D_{\rm T} & 0 & -T\chi(\rho_{\rm C})\rho^*_{\rm T} \\ 0 & 0 & D_{\rm M} & 0 \\ 0 & 0 & 0 & D_{\rm C} \end{pmatrix}, \qquad [16]$$

$$\mathbf{f}(\boldsymbol{\rho}) = \begin{pmatrix} p\,\phi(\rho_{\rm M}) - k_{\rm r}\rho_{\rm T}\rho_{\rm M} \\ k_{\rm r}\rho_{\rm T}\rho_{\rm M} - d\,\rho^*_{\rm T} \\ k_{\rm b}\rho^*_{\rm T}\rho_{\rm M}\,(1 - \rho_{\rm M}/K) - k_{\rm m}\rho_{\rm M} \\ k_{\rm e}\rho_{\rm M} - k_{\rm c}\rho_{\rm C} \end{pmatrix}. \qquad [17]$$

To understand in which parameter regimes the model spontaneously segregates cortex and medulla, we perform a linear stability analysis of the homogeneous steady state (HSS) using Wolfram Mathematica. The general procedure is as follows:

1. Determine the homogeneous steady state (HSS) that represents the stationary configuration of the system with uniform cell and chemoattractant densities. The HSS with positive densities for all components is given for the wild-type parameters (Tab. I in the main text) by $\hat\rho_{\rm T} = 3.94 \cdot 10^{-5}\mu{\rm m}^{-3}$, $\hat\rho^*_{\rm T} = 3.63 \cdot 10^{-6}\mu{\rm m}^{-3}$, $\hat\rho_{\rm M} = 6.14 \cdot 10^{-5}\mu{\rm m}^{-3}$, $\hat\rho_{\rm C} = 85.98{\rm nM}$.
2. Linearize Eq. Eq. (15) around this HSS for small perturbations $\delta\rho_i = \rho_i(\mathbf{x}, t) - \hat\rho_i$, $i = {\rm T}, {\rm T}^*, {\rm M}, {\rm C}$.
3. Solve the linearized system using a normal mode decomposition. This yields the growth rates σ^i_q, $i = 1, 2, 3, 4$ of the normal modes with mode number q. These indicate which perturbations around the HSS grow or decay (see below).

The HSS densities $\boldsymbol{\rho}_{\rm hss}$ (given above for the wild-type parameters) are found by solving $0 = \mathbf{f}(\boldsymbol{\rho}_{\rm hss})$ because all gradients vanish in the case of uniform densities. The linearized dynamics are obtained by inserting $\boldsymbol{\rho} = \boldsymbol{\rho}_{\rm hss} + \delta\boldsymbol{\rho}$ in Eq. Eq. (15) and keeping only those terms linear in $\delta\boldsymbol{\rho}$. This yields

$$\partial_t \delta\boldsymbol{\rho} = \left[\mathbf{M}(\boldsymbol{\rho}_{\rm hss})\nabla^2 + \mathbf{J}\right]\delta\boldsymbol{\rho}\,, \qquad [18]$$

with the Jacobian

$$J_{ij} = \partial_{\rho_j} f_i(\boldsymbol{\rho})\big|_{\boldsymbol{\rho}=\boldsymbol{\rho}_{\rm hss}}\,. \qquad [19]$$

The linearized dynamics, Eq. 18, together with the periodic boundary conditions, can then be solved using a normal mode decomposition (Fourier series)

$$\delta\boldsymbol{\rho}(\mathbf{x},t) = \sum_{\mathbf{q}} e^{\sigma_q t} e^{i\mathbf{q}\mathbf{x}} \delta\boldsymbol{\rho}_{\mathbf{q}} \,. \tag{20}$$

Here, the growth rate σ_q for a Fourier mode with wavenumber $q = |\mathbf{q}|$ solves the eigenvalue equation

$$\sigma_q \delta\boldsymbol{\rho}_{\mathbf{q}} = \left[-q^2 \mathbf{M}(\boldsymbol{\rho}_{\mathrm{hss}}) + \mathbf{J}\right] \delta\boldsymbol{\rho}_{\mathbf{q}} \,. \tag{21}$$

This eigenvalue equation for the growth rates σ_q can be solved employing Wolfram Mathematica's `Eigenvalue[]` function for the matrix $\left[-q^2 \mathbf{M}(\boldsymbol{\rho}_{\mathrm{hss}}) + \mathbf{J}\right]$. Solving this eigenvalue equation, one obtains four growth rates σ_q^i with $i = 1, 2, 3, 4$ because the diagonalized matrix is four-dimensional. The real part of the eigenvalues indicates whether the amplitude of the corresponding perturbation mode grows ($\mathrm{Re}[\sigma_q^i] < 0$) or decays ($\mathrm{Re}[\sigma_q^i] > 0$) [cf. Eq. Eq. (20)]. To analyze the stability of the HSS, one defines the dispersion relation σ_q as the growth rate with the maximal real part for a given wavenumber q.

For parameter values for which the real part of the growth rates is negative for all wavenumbers q, $\mathrm{Re}[\sigma_q] < 0$, the HSS is linearly stable. In contrast, in the case that $\mathrm{Re}[\sigma_q] > 0$ holds for a band of wavenumbers $q \in (q_-, q_+)$ with $q_+ > q_- \geq 0$, small deviations from the HSS with wavenumbers $q \in (q_-, q_+)$ will grow over time and the HSS is unstable. Because the HSS is only unstable due to the growth of spatially inhomogenenous perturbations, this instability is called a lateral instability. For the analyzed parameter region, one finds $q_- > 0$ because the HSS is locally stable, i.e., $\mathrm{Re}[\sigma_0] < 0$. Consequently, the lateral instability is of type I within the classification scheme of Cross and Hohenberg (84). Figure 4(A) in the main text shows the resulting parameter regime in which the system is laterally unstable (gray-shaded region).

We now discuss the two mechanisms underlying this lateral instability in the spatial cross-talk model. To this end, we study two reduced models that either exclude chemotaxis or the reaction–diffusion instability.

B. Chemotaxis-free model. Figure 4(A–B) of the main text shows that the spatial cross-talk model predicts that the thymus tissue segregates into cortex and medulla even without chemotaxis. To analyze the lateral instability in this case [blue-shaded parameter region in Fig. 4(A)], we set the chemotaxis strength T in Eqs. Eq. (14) to zero which yields the chemotaxis-free system

$$\partial_t \rho_{\mathrm{T}}(\mathbf{x},t) = D_{\mathrm{T}} \nabla^2 \rho_{\mathrm{T}} - k_{\mathrm{r}} \rho_{\mathrm{T}} \rho_{\mathrm{M}} + p\,\phi(\rho_{\mathrm{M}}) \,, \tag{22a}$$

$$\partial_t \rho_{\mathrm{T}}^*(\mathbf{x},t) = D_{\mathrm{T}} \nabla^2 \rho_{\mathrm{T}}^* + k_{\mathrm{r}} \rho_{\mathrm{T}} \rho_{\mathrm{M}} - d\,\rho_{\mathrm{T}}^* \,, \tag{22b}$$

$$\partial_t \rho_{\mathrm{M}}(\mathbf{x},t) = D_M \nabla^2 \rho_{\mathrm{M}} + k_{\mathrm{b}} \rho_{\mathrm{T}}^* \rho_{\mathrm{M}} \left(1 - \frac{\rho_{\mathrm{M}}}{K}\right) - k_{\mathrm{m}} \rho_{\mathrm{M}} \,. \tag{22c}$$

This model contains a positive feedback loop based on the growth and decay of mTECs (cf. Sec. III.A in the main text), which we show explicitly to induce a reaction–diffusion (Turing) instability in this section. As discussed in the main text, the feedback loop is due to the increased production of activated thymocytes in regions of increased mTEC densities by recognition (first reaction term in Eq. Eq. (22b)). In return, the activated thymocytes stimulate further proliferation of the mTECs, enhancing initial variations in the mTEC density (first reaction term in Eq. Eq. (22c)).

To simplify the feedback mediated by the production of activated thymocytes, we will neglect their time evolution. We assume that the density of activated thymocytes adapts instantaneously to changes in the densities of the mTECs and the non-activated thymocytes. Thus, we approximate the density ρ_{T}^* by its quasi-steady-state (QSS) distribution $\tilde{\rho}_{\mathrm{T}}^*$ obtained by setting $\partial_t \tilde{\rho}_{\mathrm{T}}^* = 0$ in Eq. Eq. (22b). While this approximation does not give the exact temporal dynamics of the system, it does not change the steady-state patterns.

To calculate the QSS density distribution, we define the linear operator $\mathcal{L}^{-1} \equiv (1 - D_{\mathrm{T}}/d\,\nabla^2)^{-1}$.* Then, starting from Eq. Eq. (22b) and setting $\partial_t \tilde{\rho}_{\mathrm{T}}^* = 0$, one obtains the QSS distribution of the activated thymocytes $\tilde{\rho}_{\mathrm{T}}^*$ as

$$\tilde{\rho}_{\mathrm{T}}^* = \frac{k_{\mathrm{r}}}{d} \mathcal{L}^{-1} \rho_{\mathrm{T}} \rho_{\mathrm{M}}. \tag{23}$$

Note that the operator $\mathcal{L}^{-1}$ is the identity when acting on uniform densities. Therefore, to separate its uniform contribution from the one depending on gradients in the density fields $\rho_{\mathrm{T,M}}$, we rewrite the operator as

$$\mathcal{L}^{-1} \equiv 1 + \frac{D_{\mathrm{T}}}{d} \mathcal{D}. \tag{24}$$

Here, the linear operator $\mathcal{D}$ defined by this equation can be expanded for weak gradients as $\mathcal{D} = \nabla^2 + \mathcal{O}(\nabla^4)$. Thus, $\mathcal{D}$ can be understood as a generalized diffusion operator that approaches the Laplacian ∇^2 in the long-wavelength limit. Inserting the

*The linear operator $\mathcal{L}^{-1}$ acts as the convolution with a Yukawa kernel. Given a function $g(\mathbf{x})$ for $\mathbf{x} \in \mathbb{R}^3$, one has $\mathcal{L}^{-1} g = \int \mathrm{d}^3 y K(|\mathbf{x} - \mathbf{y}|) g(\mathbf{y})$ with $K(r) = \exp(-r/\sqrt{D_{\mathrm{T}}/d})/r$.

quasi-steady state distribution Eq. Eq. (23) into the mTEC dynamics Eq. Eq. (22c), one obtaines the reduced system

$$\partial_t \rho_{\mathrm{T}}(\mathbf{x},t) = D_{\mathrm{T}} \nabla^2 \rho_{\mathrm{T}} - k_{\mathrm{r}} \rho_{\mathrm{T}} \rho_{\mathrm{M}} + p\,\phi(\rho_{\mathrm{M}})\,, \tag{25a}$$

$$\begin{aligned}\partial_t \rho_{\mathrm{M}}(\mathbf{x},t) &= D_{\mathrm{M}} \nabla^2 \rho_{\mathrm{M}} + \frac{D_{\mathrm{T}} k_{\mathrm{b}} k_{\mathrm{r}}}{d^2} \rho_{\mathrm{M}} \left(1 - \frac{\rho_{\mathrm{M}}}{K}\right) \mathcal{D} \rho_{\mathrm{T}} \rho_{\mathrm{M}} \\ &\quad + \frac{k_{\mathrm{b}} k_{\mathrm{r}}}{d} \rho_{\mathrm{T}} \rho_{\mathrm{M}}^2 \left(1 - \frac{\rho_{\mathrm{M}}}{K}\right) - k_{\mathrm{m}} \rho_{\mathrm{M}}\,.\end{aligned} \tag{25b}$$

Because the thymocytes diffuse much more quickly than the sedentary mTECs, gradients in the mTEC density are large compared to gradients in the thymocyte density ρ_{T}, allowing one to approximate $\mathcal{D}\rho_{\mathrm{T}}\rho_{\mathrm{M}} \approx \rho_{\mathrm{T}}\mathcal{D}\rho_{\mathrm{M}}$. Using this approximation, we define the effective mTEC diffusion coefficient

$$D_{\mathrm{M}}^{\mathrm{eff}}(\rho_{\mathrm{T}}, \rho_{\mathrm{M}}) \equiv \frac{D_{\mathrm{T}} k_{\mathrm{b}} k_{\mathrm{r}}}{d^2} \rho_{\mathrm{T}} \rho_{\mathrm{M}} \left(1 - \frac{\rho_{\mathrm{M}}}{K}\right). \tag{26}$$

Linearizing around the homogeneous steady state $\boldsymbol{\rho}_{\mathrm{hss}}$, which is unaffected by the approximations performed in this section, the diffusion coefficient $D_{\mathrm{M}}^{\mathrm{eff}}(\rho_{\mathrm{T}}, \rho_{\mathrm{M}})$ is set constant to its value $D_{\mathrm{M}}^{\mathrm{eff}} \equiv D_{\mathrm{M}}^{\mathrm{eff}}(\rho_{\mathrm{T,hss}}, \rho_{\mathrm{M,hss}})$ evaluated at the densities $\boldsymbol{\rho}_{\mathrm{hss}}$. We then arrive at the generalized two-component reaction–diffusion system

$$\partial_t \rho_{\mathrm{T}}(\mathbf{x},t) = D_{\mathrm{T}} \nabla^2 \rho_{\mathrm{T}} - k_{\mathrm{r}} \rho_{\mathrm{T}} \rho_{\mathrm{M}} + p\,\phi(\rho_{\mathrm{M}})\,, \tag{27a}$$

$$\begin{aligned}\partial_t \rho_{\mathrm{M}}(\mathbf{x},t) &= \left[D_{\mathrm{M}} \nabla^2 + D_{\mathrm{M}}^{\mathrm{eff}} \mathcal{D}\right] \rho_{\mathrm{M}} \\ &\quad + \frac{k_{\mathrm{b}} k_{\mathrm{r}}}{d} \rho_{\mathrm{T}} \rho_{\mathrm{M}}^2 \left(1 - \frac{\rho_{\mathrm{M}}}{K}\right) - k_{\mathrm{m}} \rho_{\mathrm{M}}\,.\end{aligned} \tag{27b}$$

In this system, the thymocytes (density ρ_{T}) are produced and degraded based on the mTEC density ρ_{M}. In turn, the mTEC density decays linearly due to cell death ($\sim k_{\mathrm{m}}$) but has a T-cell dependent second-order self-production term $\sim \rho_{\mathrm{T}}\rho_{\mathrm{M}}^2$ which stems from the feedback loop, i.e., the local part of $\tilde{\rho}_{\mathrm{T}}^*$ [cf. Eqs. Eq. (23), Eq. (24)]. The non-local part of $\tilde{\rho}_{\mathrm{T}}^*$, i.e., the diffusion of the activated thymocytes, reduces to the additional diffusion term with the effective diffusion coefficient $D_{\mathrm{M}}^{\mathrm{eff}}$ for the mTECs. While the reactions give rise to a stable fixed point $\mathrm{Re}[\sigma_0] < 0$, the system is laterally unstable due to a Turing instability (85). This Turing instability is possible because the thymocytes diffuse much more quickly than the mTECs as the effective diffusion constant $D_{\mathrm{M}}^{\mathrm{eff}}$ is about two orders of magnitude smaller than D_{T} (D_{M} is even smaller). While the two-component system Eqs. Eq. (27) is not an exact reaction–diffusion system due to the generalized diffusion operator $\mathcal{D}$, in the long-wavelength limit, one can use $\mathcal{D} \approx \nabla^2$ to arrive at

$$\partial_t \rho_{\mathrm{T}}(\mathbf{x},t) = D_{\mathrm{T}} \nabla^2 \rho_{\mathrm{T}} - k_{\mathrm{r}} \rho_{\mathrm{T}} \rho_{\mathrm{M}} + p\,\phi(\rho_{\mathrm{M}})\,, \tag{28a}$$

$$\begin{aligned}\partial_t \rho_{\mathrm{M}}(\mathbf{x},t) &= \left(D_{\mathrm{M}} + D_{\mathrm{M}}^{\mathrm{eff}}\right) \nabla^2 \rho_{\mathrm{M}} \\ &\quad + \frac{k_{\mathrm{b}} k_{\mathrm{r}}}{d} \rho_{\mathrm{T}} \rho_{\mathrm{M}}^2 \left(1 - \frac{\rho_{\mathrm{M}}}{K}\right) - k_{\mathrm{m}} \rho_{\mathrm{M}}\,.\end{aligned} \tag{28b}$$

Figure S1 shows the dispersion relations σ_q obtained for the wild-type parameters with the full chemotaxis-free model Eq. Eq. (22), the reduced model with generalized diffusion Eq. Eq. (27), and the complete reduction to the Turing system Eq. Eq. (28). All systems show a band of unstable modes. The QSS approximation (and $\mathcal{D}\rho_{\mathrm{T}}\rho_{\mathrm{M}} \approx \rho_{\mathrm{T}}\mathcal{D}\rho_{\mathrm{M}}$) only introduces small deviations in the dispersion relation. The reduced reaction–diffusion system Eqs. Eq. (28) approximates the dispersion relation of the full system well at long wavelengths (small wavenumbers q) while it deviates strongly at shorter wavelength due to neglecting higher-order gradient terms in the approximation $\mathcal{D} \approx \nabla^2$. Importantly, the reduced system Eqs. Eq. (28) retains the emergence of a band of unstable modes q with $\sigma_q > 0$ above a minimal wavenumber q_- while the system is locally stable ($\sigma_0 < 0$). This verifies that the lateral instability of the full chemotaxis-free model is a generalization of the instability found in the reduced two-component system.

Two-component reaction–diffusion systems show a Turing instability if the Segel–Jackson criterion is fulfilled (86). It requires that the components of the Jacobian of the reaction terms at the hss have a particular combination of signs. The slow-diffusing species must have a positive, the fast-diffusing species a negative diagonal entry. Moreover, the off-diagonal terms must have opposite signs. The linearization of Eq. Eq. (28) gives the Jacobian of the reaction terms

$$\mathbf{J}_{\mathrm{reac}} = \begin{pmatrix} -k_{\mathrm{r}}\rho_{\mathrm{M}} & -k_{\mathrm{r}}\rho_{\mathrm{T}} + p\partial_{\rho_{\mathrm{M}}}\phi \\ \frac{k_{\mathrm{b}}k_{\mathrm{r}}}{d}\rho_{\mathrm{M}}^2\left(1 - \frac{\rho_{\mathrm{M}}}{K}\right) & 2\frac{k_{\mathrm{b}}k_{\mathrm{r}}}{d}\rho_{\mathrm{T}}\rho_{\mathrm{M}}\left(1 - \frac{\rho_{\mathrm{M}}}{K}\right) - k_{\mathrm{m}} \end{pmatrix} \tag{29}$$

with $\partial_{\rho_{\mathrm{M}}}\phi < 0$ and $\rho_{\mathrm{M}} < K$. Thus, the signs of the components are

$$\begin{pmatrix} - & - \\ + & +/- \end{pmatrix}. \tag{30}$$

The second diagonal term becomes positive if the feedback term $\sim \rho_{\mathrm{T}}\rho_{\mathrm{M}}^2$ becomes dominant. Then, the system can undergo a Turing instability. The resulting combination of signs in the Jacobian is called an activator–substrate system or depletion model (87, 88).

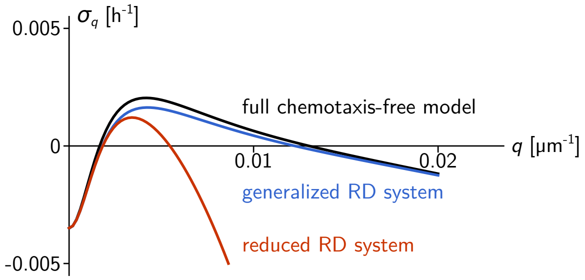

Fig. S1. Dispersion relation of the chemotaxis-free model. Comparison of the dispersion relations of the chemotaxis-free model Eq. Eq. (22) (black), the reduced generalized reaction–diffusion system Eq. Eq. (27) (blue), and the reduced reaction–diffusion system Eq. Eq. (28) obtained in the long-wavelength limit (red). The dispersion relations are calculated for the wild-type parameters given in Table I.

Taken together, the reduced two-component reaction–diffusion system Eqs. Eq. (28) shows that the feedback mediated by activated thymocytes allows for a Turing instability in the system. This instability drives the segregation of cortex and medulla in the spatial thymic cross-talk model if the thymocytes do not undergo chemotaxis.

Instability mechanism for $D_\mathrm{T} \gg D_\mathrm{M}^\mathrm{eff}$ — For the wild-type parameters, one has $D_\mathrm{T} \gg D_\mathrm{M} + D_\mathrm{M}^\mathrm{eff}$ (by two orders of magnitude). In this case, one can simplify the linear stability analysis by taking the limit $(D_\mathrm{M} + D_\mathrm{M}^\mathrm{eff})/D_\mathrm{T} \to 0$. Then, due to their fast diffusion, the thymocyte density ρ_T can be assumed as uniform on the length scales on which the mTEC density varies. Assuming the thymocyte density as constant, the linearized mTEC dynamics Eq. Eq. (28b) read

$$\partial_t \delta\rho_\mathrm{M}(\mathbf{x}, t) = \left(D_\mathrm{M} + D_\mathrm{M}^\mathrm{eff}\right) \nabla^2 \delta\rho_\mathrm{M} + \left(\partial_{\rho_\mathrm{M}} \tilde{f}_\mathrm{M}(\boldsymbol{\rho}_\mathrm{hss})\right) \delta\rho_\mathrm{M}, \qquad [31]$$

with $\tilde{f}_\mathrm{M}(\boldsymbol{\rho}) = (k_\mathrm{b} k_\mathrm{r}/d)\rho_\mathrm{T}\rho_\mathrm{M}^2(1-\rho_\mathrm{M}/K) - k_\mathrm{m}\rho_\mathrm{M}$. Thus, the reaction term is the lower diagonal entry of the full Jacobian Eq. Eq. (29). The growth rates σ_q for $q > 0$ read

$$\sigma_q = \left(\partial_{\rho_\mathrm{M}} \tilde{f}_\mathrm{M}(\boldsymbol{\rho}_\mathrm{hss})\right) - \left(D_\mathrm{M} + D_\mathrm{M}^\mathrm{eff}\right) q^2, \qquad [32]$$

and positive growth rates only occur if

$$\partial_{\rho_\mathrm{M}} \tilde{f}_\mathrm{M}(\boldsymbol{\rho}_\mathrm{hss}) > 0\,, \qquad [33]$$

i.e., if the lower diagonal term of the Jacobian is positive. This is the same condition as we obtained from the Segel–Jackson criterion.

As $\tilde{f}_\mathrm{M}(\boldsymbol{\rho}_\mathrm{hss}) = 0$, an instability occurs only if $\tilde{f}_\mathrm{M}$ has a positive slope at one of its roots. The steady state of the full system corresponds to the middle root of $\tilde{f}_\mathrm{M}$ as a function of ρ_M for sufficiently small mTEC proliferation rate k_b [see Fig. S2(A)]. Indeed, $\tilde{f}_\mathrm{M}$ a function of ρ_M has a positive slope at this root because the positive feedback in mTEC proliferation—mTEC growth $\sim \rho_\mathrm{T}\rho_\mathrm{M}^2$—induces a cubic shape of $\tilde{f}_\mathrm{M}$ as a function of ρ_M. Perturbations in the mTEC density around the HSS grow because of the feedback induced by the activated thymocytes: At densities $\rho_\mathrm{M} < \rho_\mathrm{M,hss}$, the decay of mTECs dominates while proliferation dominates due to the feedback at $\rho_\mathrm{M} > \rho_\mathrm{M,hss}$ (but $\rho_\mathrm{M,hss} \lesssim K$).

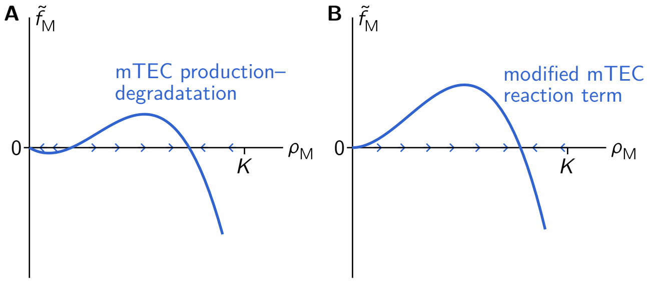

Fig. S2. The local mTEC dynamics. (A) The production and degradation term $\tilde{f}_\mathrm{M}$ of the mTEC density is shown (blue; for constant thymocyte density ρ_T). The arrows denote whether the reaction term $\tilde{f}_\mathrm{M}$ leads to an increase or decrease of of the mTEC density. (B) The modified production and degradation term is shown in which the linear mTEC death term $-k_\mathrm{m}\rho_\mathrm{M}$ is replaced by the quadratic term $-k_\mathrm{m}\rho_\mathrm{M}^2/K$.

C. Reduced model without Turing instability. In the previous section, we showed that the spatial thymic cross-talk model leads to the segregation into cortex and medulla even without chemotaxis. In this section, we modify the reaction term to exclude this chemotaxis-free (Turing) instability. We then reduce this modified system to a two-component system and show that one obtains Keller–Segel dynamics. Keller–Segel systems also show a lateral instability. Their instability is due to chemotactic

aggregation, which shows that the spatial thymic cross-talk model has a Keller–Segel-type chemotactic instability in addition to the Turing instability discussed in the previous section.

To exclude the Turing instability in the reduced two-component reaction–diffusion system Eqs. Eq. (28), one has to ensure that the second diagonal term of the Jacobian Eq. Eq. (29) is negative at the fixed point. Thus, one has to modify the reaction term $\tilde{f}_\mathrm{M}$ such that it does not have a root with positive slope at a density $\rho_\mathrm{M} > 0$. In order to not modify the interaction between thymocytes and mTECs, we will only change the mTEC degradation term $k_\mathrm{m}\rho_\mathrm{M}$. The cubic shape of the reaction term $\tilde{f}_\mathrm{M}$ is then lost if we replace the linear mTEC degradation term $-k_\mathrm{m}\rho_\mathrm{M}$ by the quadratic term $-k_\mathrm{m}\rho_\mathrm{M}^2/K$ [see Fig. S2(B)]. Then, mTEC degradation does not dominate at low densities ρ_M while the feedback-induced proliferation dominates at larger densities. Instead, the middle root of $\tilde{f}_\mathrm{M}$ is moved to zero.[†]

As a "minimal" modification, we multiply the mTEC decay term with ρ_M/K, using the carrying capacity K to nondimensionalize the density. With this modification, we arrive at the modified model without Turing instability (cf. Eqs. Eq. (14))

$$\partial_t\rho_\mathrm{T} = D_\mathrm{T}\nabla^2\rho_\mathrm{T} - \nabla(\rho_\mathrm{T}v_\mathrm{C}) - k_\mathrm{r}\rho_\mathrm{T}\rho_\mathrm{M} + p\,\phi\,, \quad [34a]$$

$$\partial_t\rho_\mathrm{T}^* = D_\mathrm{T}\nabla^2\rho_\mathrm{T}^* - \nabla(\rho_\mathrm{T}^*v_\mathrm{C}) + k_\mathrm{r}\rho_\mathrm{T}\rho_\mathrm{M} - d\,\rho_\mathrm{T}^*\,, \quad [34b]$$

$$\partial_t\rho_\mathrm{M} = D_M\nabla^2\rho_\mathrm{M} + k_\mathrm{b}\rho_\mathrm{T}^*\rho_\mathrm{M}\left(1 - \frac{\rho_\mathrm{M}}{K}\right) - k_\mathrm{m}\frac{\rho_\mathrm{M}^2}{K}\,, \quad [34c]$$

$$\partial_t\rho_\mathrm{C} = D_\mathrm{C}\nabla^2\rho_\mathrm{C} + k_\mathrm{e}\rho_\mathrm{M} - k_\mathrm{c}\rho_\mathrm{C}\,. \quad [34d]$$

The linear stability analysis of this model yields the orange-shaded instability region in Fig. 4(A) in the main text. The system is only laterally unstable if the chemotaxis-strength is finite. This shows that the instability is due to the chemotaxis of the thymocytes.

Effective Keller–Segel dynamics — The chemotaxis-driven instability is due to feedback in the chemotactic accumulation of thymocytes: (Activated) thymocytes stimulate mTEC growth. The mTECs produce chemokines that attract more thymocytes to this spatial region. Thus, thymocytes accumulate in this spatial region. The classical Keller–Segel model is the prototypical model showing this chemotaxis-driven instability (89, 90). Here, we will again use a QSS approximation to reduce the dynamics of the modified model Eqs. Eq. (34) to a Keller–Segel model with production and degradation (91).

The Keller–Segel model is a two-component system that models the density of the chemotactic cell density and the density of the chemoattractant, which is produced in dependence on the chemotactic cell density. In the modified model system Eqs. Eq. (34), the chemotactic cell density is the total thymocyte density $\rho_\mathrm{T}^\mathrm{tot} = \rho_\mathrm{T} + \rho_\mathrm{T}^*$. The thymocytes lead to chemoattractent production indirectly by the stimulation of mTEC growth. To approximate this indirect chemoattractant production by a single chemoattractant production term $p_\mathrm{C}(\rho_\mathrm{T}^\mathrm{tot})$, we determine the local QSS approximations $\tilde{\rho}_\mathrm{M}(\rho_\mathrm{T}^*)$ and $\tilde{\rho}_\mathrm{T}^*(\rho_\mathrm{T}^\mathrm{tot})$, neglecting the diffusion and chemotaxis of $\rho_\mathrm{M}, \rho_\mathrm{T}^*$. As the mTECs diffuse very slowly compared to the thymocytes, neglecting their diffusion term will only introduce minor deviations in the chemoattractant production. Neglecting diffusion and chemotaxis of the activated thymocytes ρ_T^* is an uncontrolled approximation, but it will qualitatively capture that, locally, a certain fraction of the thymocytes is activated and those stimulate mTEC proliferation, resulting in an increased chemokine production in regions of high thymocyte density.

The QSS approximation of the mTEC density ρ_M neglecting their diffusion, i.e., setting $D_\mathrm{M} = 0$) is obtained by assuming that the mTEC density has fully relaxed to its steady state. As their time evolution vanishes in this (quasi) steady state, it is obtained by setting $\partial_t\rho_\mathrm{M} = 0$ in Eq. 34c. This yields[‡]

$$\tilde{\rho}_\mathrm{M}(\rho_\mathrm{T}^*) = K\frac{\rho_\mathrm{T}^*}{\frac{k_\mathrm{m}}{k_\mathrm{b}} + \rho_\mathrm{T}^*}. \quad [35]$$

Thus, the QSS mTEC density increases with the density of activated thymocytes (as they stimulate mTEC proliferation) up to the carrying capacity K. Inserting this expression into Eq. 34b, the local QSS approximation of the activated thymocytes ρ_T^* is obtained analogously by setting $\partial_t\rho_\mathrm{T}^* = 0$ (and neglecting the gradient terms). This gives

$$\tilde{\rho}_\mathrm{T}^*(\rho_\mathrm{T}^\mathrm{tot}) = \frac{k_\mathrm{r}K}{d + k_\mathrm{r}K}\left(\rho_\mathrm{T}^\mathrm{tot} - \frac{k_\mathrm{m}d}{k_\mathrm{b}k_\mathrm{r}K}\right) \quad [36]$$

if $\rho_\mathrm{T}^\mathrm{tot} > k_\mathrm{m}d/(k_\mathrm{b}k_\mathrm{r}K)$ and zero otherwise. As a result, the density of activated thymocytes increases linearly with the total thymocyte density if $\rho_\mathrm{T}^\mathrm{tot} > k_\mathrm{m}d/(k_\mathrm{b}k_\mathrm{r}K)$.

Both relations together yield the monotonously increasing relation $\tilde{\rho}_\mathrm{M}(\rho_\mathrm{T}^\mathrm{tot})$, which results in the two-component Keller–Segel model with thymocyte production and degradation (cf. Ref. (91))

$$\partial_t\rho_\mathrm{T}^\mathrm{tot} = D_\mathrm{T}\nabla^2\rho_\mathrm{T}^\mathrm{tot} - \nabla(\rho_\mathrm{T}^\mathrm{tot}v_\mathrm{C}) + p\,\tilde{\phi}(\rho_\mathrm{T}^\mathrm{tot}) - d\,\tilde{\rho}_\mathrm{T}^*(\rho_\mathrm{T}^\mathrm{tot})\,, \quad [37a]$$

$$\partial_t\rho_\mathrm{C} = D_\mathrm{C}\nabla^2\rho_\mathrm{C} + k_\mathrm{e}\tilde{\rho}_\mathrm{M}(\rho_\mathrm{T}^\mathrm{tot}) - k_\mathrm{c}\rho_\mathrm{C}\,, \quad [37b]$$

[†] We assume that the feedback term dominates over the quadratic degradation term. Otherwise, the mTEC density is driven to zero everywhere because one would have $\tilde{f}_\mathrm{M} < 0$ for all $\rho_\mathrm{M} > 0$ (see Fig. S2(B)).

[‡] Note that this reduction is not possible without the modification of the reaction term that excludes the Turing instability. Without the modification, $\tilde{f}_\mathrm{M}$ one root more, which is the middle root with a positive slope that gives rise to the Turing instability. This additional root introduces an effective bistability of the mTEC dynamics because low-density regions of the mTEC pattern relax toward the root $\rho_\mathrm{M} = 0$ while high-density regions relax toward the third, high-density root of $\tilde{f}_\mathrm{M}$ [see Fig. S2]. The reason is that both the root at $\rho_\mathrm{M} = 0$ and the high-density root are stable in the original mTEC dynamics (keeping all other fields fixed). In contrast, only the high-density root is stable in the modified dynamics. Thus, the QSS mTEC density is given by the single (stable) root $\tilde{\rho}_\mathrm{M}(\rho_\mathrm{T}^*)$. Without the modification of the reaction term, one would have to consider different roots for different regions of the pattern.

where we defined $\tilde{\phi}(\rho_T^{tot}) = \phi(\tilde{\rho}_M(\rho_T^{tot}))$. As the production of the chemoattractant ρ_C increases with the local density of the thymocytes, this system allows for chemotaxis-driven accumulation of the thymocytes.[§]

To summarize the linear stability analysis, this mathematical analysis allows to determine the parameter region in which the model system spontaneously segregrates into cortex and medulla. We then constructed two reduced mathematical models that either only show a reaction–diffusion or only a chemotaxis-driven instability. Using QSS approximations, we reduced these two model systems to two-component systems. The two-component reaction–diffusion system shows that the reaction–diffusion (Turing) instability is driven by the positive feedback in mTEC proliferation mediated by the activated thymocytes. In contrast, the two-component system for the chemotaxis-driven instability mechanism resembles the Keller–Segel model, clarifying that its instability is driven by the self-amplifying chemotactic aggregation of the thymocytes. In the full spatial thymic cross-talk model, both instability mechanisms play together, resulting in the increased unstable region of the full model (gray-shaded region) compared to the instability regions of the single mechanisms (blue- and orange-shaded region) shown in Fig. 4(A) in the main text.

D. Characteristic wavelength of the linear instability. If the homogeneous steady state is unstable due to a lateral instability, the real part of the growth rates $\mathrm{Re}(\sigma_q)$, that is, the dispersion relation determines how fast perturbations with different wavelength grow. Because the fastest-growing mode with the largest real part of the growth rate outgrows modes with different wavenumbers, the wavelength of the fastest-growing mode determines the characteristic wavelength of the pattern initially growing out of the homogeneous steady state. As the pattern grows in amplitude, nonlinear effects become relevant and may lead to changes in the wavelength of the fully-grown large-amplitude pattern.

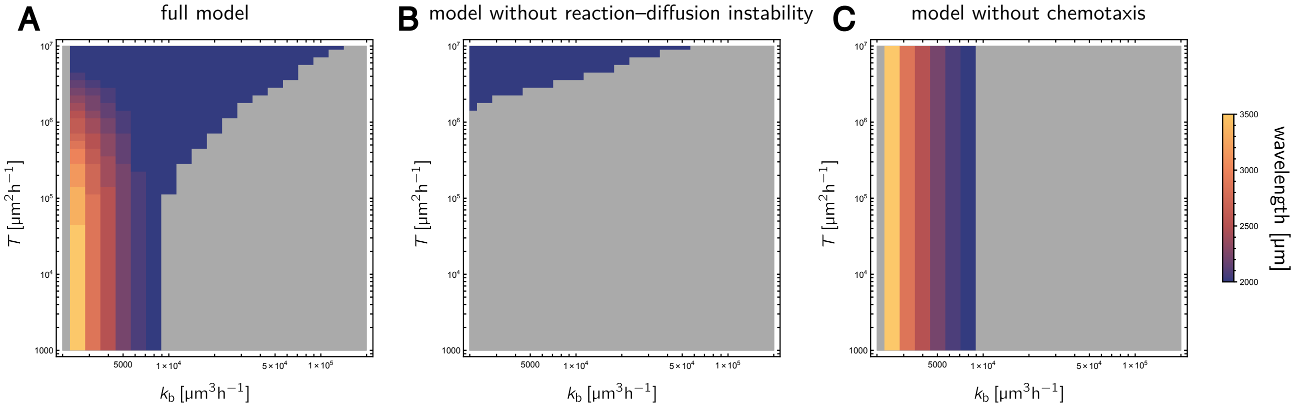

Fig. S3. The wavelengths of the fastest-growing modes. The fastest-growing mode of the linear instability has the largest real part of the growth rate. Its wavelength is shown for the full spatial thymic cross-talk model (A), the reduced model without Turing instability, Eq. Eq. (34) (B), and in the model without chemotaxis, Eq. Eq. (22) (C). The reduced models capture the wavelength of the instability in the full model at high (B) and low chemotaxis strength T (C), respectively.

These characteristic wavelengths, that is, the wavelengths of the fastest-growing mode are shown in Fig. S3. The wavelengths obtained from linear stability analysis are about two to three times as large as the length scales (corresponding to about half a wavelength, see SI Sec. B.2) measured for the fully nonlinear patterns in numerical simulations in Fig. 5 in the main text. The two reduced models, that either exclude chemotaxis (see Sec. B) or the Turing instability (see Sec. C), well describe the wavelengths of the fastest-growing modes in the spatial thymic cross-talk model at low and high chemotaxis strength, respectively.

3. Scaling of the medullary volume fraction

In the main text, we argue that the medullary volume fraction depends on the relative strength of thymocyte production in the cortex and degradation in the medulla because the (activated) thymocytes stimulate mTECs to proliferate. Thus, the total number of thymocytes is related to the amount of medullary cells. We show in Fig. 5A that this behavior is indeed observed in numerical simulations. Here, we give a formal argument to derive the scaling of the medullary volume fraction.

We assume a sharp interface between the cortex and the medulla and define the medullary and cortical volume fractions as $\varphi_{M,C}$ with $\varphi_M + \varphi_C = 1$. Production of thymocytes occurs in the cortex. From Eq. Eq. (14a) the total thymocyte production per time and total thymic volume then follows as $P_{tot} \approx p\,\varphi_C$. Moreover, activated thymocytes are degraded. Because the thymocytes are activated in the medulla, they are mainly degraded in the medulla. From Eq. Eq. (14b), we find the approximate total degradation per time and total thymic volume as $D_{tot} \approx d\langle\rho_T^*\rangle_M \varphi_M$. Here $\langle\rho_T^*\rangle_M$ is the average density of activated thymocytes in the medulla. This density will be set by the pattern-forming mechanism creating the stationary interface between

[§]Also, note that the reaction terms give rise to a Jacobian with the signs

$$\begin{pmatrix} - & 0 \\ + & - \end{pmatrix}, \qquad [38]$$

which does not fulfill the Segel–Jackson criterion and thus does not allow for a reaction–diffusion instability.

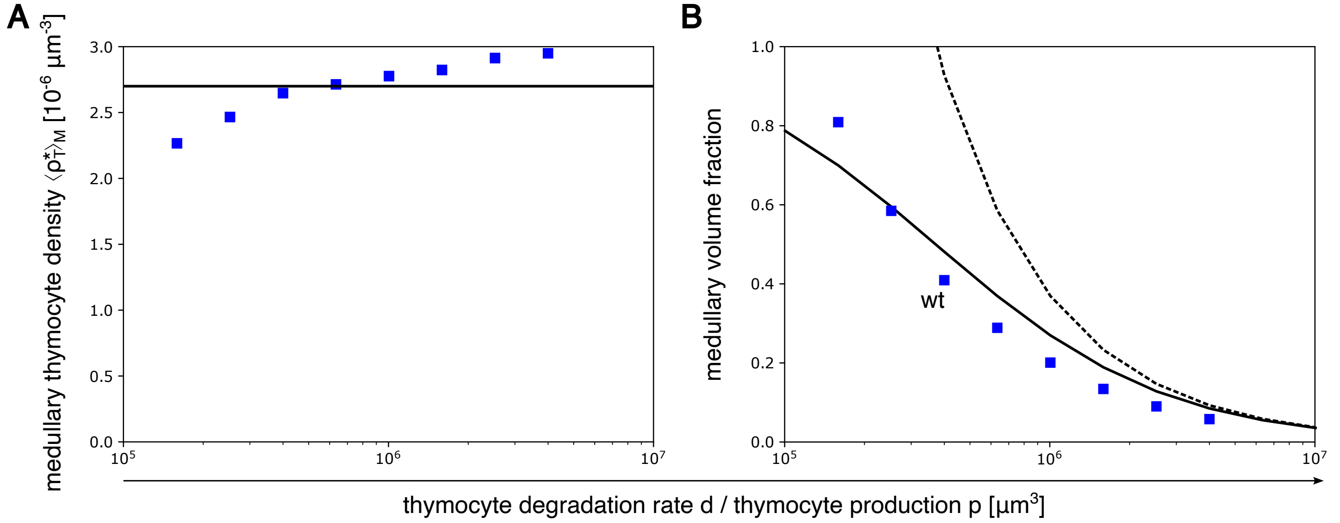

Fig. S4. The scaling of the medullary volume fraction with thymocyte production and degradation. (*A*) The average density of activated thymocytes in the medulla $\langle\rho_{\mathrm{T}}^*\rangle_{\mathrm{M}}$ varies only slowly with the ratio d/p of the thymocyte degradation rate and thymocyte production. Its average lies at approximately $2.7\cdot 10^{-6}\mathrm{\mu m}^{-3}$ (black line). (*B*) Using the averaged thymocyte density $\langle\rho_{\mathrm{T}}^*\rangle_{\mathrm{M}} \approx 2.7\cdot 10^{-6}\mathrm{\mu m}^{-3}$, the dependence of the medullary volume fraction measured in simulations (blue squares) on the ratio d/p is well predicted by Eq. Eq. (40) (black line). The approximation $\phi_{\mathrm{M}} \approx p/(d\langle\rho_{\mathrm{T}}^*\rangle_{\mathrm{M}})$ valid for small volume fractions is given as dashed line. The parameter values chosen for the wild-type simulations (wt) are marked. The simulation data corresponds to the diagonal (corresponding to keeping $p\cdot d$ fixed) intersecting the wild-type parameter set (point A) in Fig. 5 in the main text.

cortex and medulla, i.e., the balance of the fluxes of all the different components between the two domains and the balance of production and degradation of the chemoattractant creating the gradients driving chemotactic movement into the medulla (92, 93). In the limit of weak thymocyte production and degradation, we expect that their ratio only fixes the size of the cortical (production) and the medullary (degradation) region, i.e., the position of the interface. Consequently, we expect that the average cellular densities are only weakly affected by the (weak) production and degradation terms. We indeed observe numerically that $\langle\rho_{\mathrm{T}}^*\rangle_{\mathrm{M}}$ only changes slowly with p and d (see Fig. S4).

Taken together, balancing thymocyte production and degradation, $P_{\mathrm{tot}} = D_{\mathrm{tot}}$, yields

$$p(1-\varphi_{\mathrm{M}}) \approx d\langle\rho_{\mathrm{T}}^*\rangle_{\mathrm{M}}\varphi_{\mathrm{M}}. \qquad [39]$$

Thus, treating $\langle\rho_{\mathrm{T}}^*\rangle_{\mathrm{M}}$ as a constant that can be determined from numerical simulations, we predict the scaling of the medullary volume fraction as

$$\varphi_{\mathrm{M}} \approx \frac{1}{1+\frac{d}{p}\langle\rho_{\mathrm{T}}^*\rangle_{\mathrm{M}}} \approx \frac{p}{d}\frac{1}{\langle\rho_{\mathrm{T}}^*\rangle_{\mathrm{M}}}. \qquad [40]$$

Here, the last approximation holds at sufficiently small volume fractions, i.e., at $d\langle\rho_{\mathrm{T}}^*\rangle_{\mathrm{M}} \gg p$. This approximation compares well to the numerical results (see Fig. S4).

4. Numerical Simulation and Analysis

In the main text, we make use of several observables to quantify the system's behavior in numerical simulations, such as the medullary volume fraction and the length scale of the cortex-medulla pattern. In the following, we first give details on the numerical simulations and then define the observables used. Data and analysis code can be found at https://doi.org/10.5281/zenodo.15570891.

A. Numerical simulation. The model simulations are performed using the time-dependent finite-element solver of COMSOL Multiphysics (Version 6.0) (94) on a free tetrahedral (triangular) mesh with linear Lagrange elements of a maximal mesh-element size of 60µm in three, and 10µm in two dimensions. Linear stability analysis was performed using Wolfram MATHEMATICA, and analysis was performed using Python 3.6, if not indicated otherwise. Model parameters are used as specified in Tables S1, S2.

The medulla develops out of small medullary islets of monoclonal origin (95). To mirror this in our simulations, we initialize the mTEC-density ρ_{M} with small Gaussian peaks placed into the simulation volume. These initially placed ρ_{M}-peaks are subject to the same kinetics as other ρ_{M}-densities and evolve as part of the medulla. In the three-dimensional simulation, we choose Gaussian peaks with a full width at half maximum (FWHM) of 280µm as an initial condition, and to enforce periodic boundary conditions, we place a duplicate Gaussian at $x_i + L$ (or $x_i - L$) if its peak is within 300µm of the lower (or upper) simulation boundary in euclidean direction $i = 1, 2, 3$. In the two-dimensional sweep, we do not enforce periodic boundary conditions for the medulla seeds and choose a FWHM of 140µm. The solver determines initial conditions consistent with the boundary conditions from these. Because of the finer mesh chosen for these simulations, the resulting sharper gradients do not

lead to numerical artifacts. The chemokine density and all thymocyte densities are set to zero as an initial condition in all simulations.

The three-dimensional simulations show small numerical artifacts with some points showing negative mTEc-densities on the scale of $\sim -1 \cdot 10^{-6}\mu\text{m}^{-3}$. While in the most prominent case, this reaches $-9.6 \cdot 10^{-6}\mu\text{m}^{-3}$, most of the points with sub-zero densities fulfill $-2 \cdot 10^{-6}\mu\text{m}^{-3} < \rho_\text{M}$, and thus are below 2% of the density scale μ.

To ensure that these artifacts do not influence the medullary morphology we observe in our simulations, we have simulated the most affected parameter set (short length scale, Fig. 3 B, main text) with the maximal element edge length reduced by two, i.e., with a maximal element edge length of 30µm. Because it is numerically prohibitive to simulate these large-scale, three-dimensional simulations with a reduced element size at the same scale as the original simulations, we simulated a smaller volume ($L = 1500\mu\text{m}$). Crucially, this simulation displays the same medullary morphology and length scale as the original simulation, as can be seen in Fig. S5, while it does not show negative mTEC-densities after the first time step $t = 10\text{h}$. Furthermore, we calculated the volume-normalized and discretized L^2-norm of the difference of the $\rho_{\text{M,fine/coarse}}(\boldsymbol{x})$ densities in this fine simulation and a simulation of the small system with the maximal element edge length of the large simulation of 60µm, which shows negative densities. This average difference in mTEC distributions is $\Delta = \sqrt{\int \text{d}^3x\,(\rho_{\text{M,coarse}} - \rho_{\text{M,fine}})^2/L^3} \approx 6.6\% \cdot \mu$ at $t = 2000\text{h}$. Thus, we conclude that the scattered points of negative density we observe in our simulations do not influence the system's morphology and length scale.

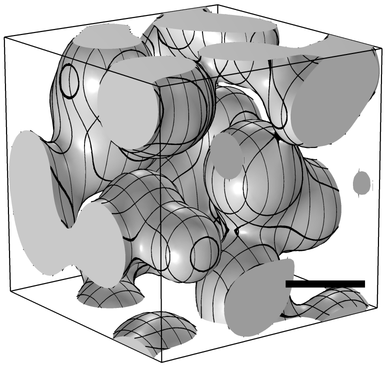

Fig. S5. Finer numerical meshing does not change medullary morphology. At a maximal element edge-length of $30\mu\text{m}$, the simulation for short medullary length scales (c.f. Fig. 3 B, main text) shows the same morphology, as well as the same length scale as the simulation for a larger domain shown in the main text with a coarser finite-element mesh at a maximal element edge length of $60\mu\text{m}$. Scale bar $500\mu\text{m}$ and domain edge length $1500\mu\text{m}$.

B. Observables.

B.1. Medullary volume fraction. One observable describing the medullary morphology is the medullary volume fraction $\varphi_\text{M}(\mathbf{x})$ (see Figs. 3, 5A in the main text). The medullary volume fraction is defined as the volume of the medulla divided by the volume of the full simulation domain. To determine its value, we define the (logistic) indicator function

$$\psi(\mathbf{x}) = \frac{1}{1 + e^{-(\rho_\text{M}(\mathbf{x}) - \mu)/10^{-6}\mu\text{m}^{-3}}} . \qquad [41]$$

The indicator function is close to zero for mTEC densities below the threshold $\rho_\text{M}(\mathbf{x}) \ll \mu$ and rapidly increases to one around $\rho_\text{M}(\mathbf{x}) = \mu = 10^{-4}\mu\text{m}^{-3}$. The small density scale $10^{-6}\mu\text{m}^{-3}$ in the exponent ensures a sharp transition between zero and one.

We average this indicator function over the simulation volume using Comsol's "Volume Average" (derived value) function. This function implements the spatial average

$$\langle \bullet \rangle \equiv \frac{1}{|\Omega|} \int_\Omega \text{d}^d x\ \bullet . \qquad [42]$$

Here, Ω is the two- ($d = 2$; used in Fig. 5A in the main text) or three-dimensional ($d = 3$; used in Fig. 3 in the main text) simulation domain with the volume $|\Omega|$. We then define the medullary volume fraction as $\varphi_\text{M} = \langle \psi \rangle$.

B.2. Pattern length scale. Moreover, the medullary pattern is quantified by its typical pattern wavelength in Fig. 5B in the main text. The pattern length scale is calculated using the connected two-point correlator for the mTEC density,

$$C(\mathbf{r}) = \langle \rho_\text{M}(\mathbf{x}) \cdot \rho_\text{M}(\mathbf{x} + \mathbf{r}) \rangle - \langle \rho_\text{M}(\mathbf{x}) \rangle^2 , \qquad [43]$$

using the average Eq. Eq. (42).

Because the system dynamics are isotropic (up to the boundary conditions), we expect that the correlation function depends only in the distance $r = |\mathbf{r}|$. We numerically calculated this radial correlation function $C(r)$ by averaging over all directions using Python (with a different normalization of the average $\langle \bullet \rangle$ that will be irrelevant for the following). From the radial correlation function $C(r)$, we determine its first minimum, whose value r_0 of the distance coordinate r gives the typical length scale on which the pattern is anti-correlated. Thus, r_0 can be interpreted as half of the typical pattern length scale. To account for noise in the data that might produce local minima in an otherwise falling function, we determine the minimum by the following algorithm. We search for the minimum in a sliding window of 11 data points which we iteratively center around the newly found minimum. Once the minimum of the window is in its center, the iteration stops. We then determine the minimum by fitting a quadratic function to the 11 points in the window. If no minimum is found using this method, the length scale is set to the length associated with the correlator's absolute minimum.

B.3. Thymocyte localization. Fig. 4*D, E* in the main text analyzes the localization of the thymocytes in the medullary structure. Specifically, we measure the local density of thymocytes within the medulla. Therefore, we define the average thymocyte density in the medulla $\langle \rho_\mathrm{T}(\mathbf{x}) \rangle_\mathrm{M}$ as

$$\langle \rho_\mathrm{T}(\mathbf{x}) \rangle_\mathrm{M} \equiv \frac{\langle \psi(\mathbf{x}) \rho_\mathrm{T}(\mathbf{x}) \rangle}{\langle \psi(\mathbf{x}) \rangle} \,. \qquad [44]$$

Moreover, the average thymocyte density in the cortex $\langle \rho_\mathrm{T}(\mathbf{x}) \rangle_\mathrm{C}$ is defined as

$$\langle \rho_\mathrm{T}(\mathbf{x}) \rangle_\mathrm{C} \equiv \frac{\langle (1-\psi(\mathbf{x})) \rho_\mathrm{T}(\mathbf{x}) \rangle}{\langle (1-\psi(\mathbf{x})) \rangle} \,. \qquad [45]$$

The values of these two averages in the simulations are plotted as bar charts in Fig. 4*D, E* in the main text.

Furthermore, to give a quantitative measure for thymocyte-medulla colocalization, we use the covariance of the self-reactive and tolerant thymocyte species ρ_T and ρ_Tol, with the mTEC densities ρ_M, which we normalize with their respective volume-averaged mean,

$$C_\mathrm{T,M} = \frac{\langle \rho_\mathrm{T} \rho_\mathrm{M} \rangle - \langle \rho_\mathrm{T} \rangle \langle \rho_\mathrm{M} \rangle}{\langle \rho_\mathrm{T} \rangle \langle \rho_\mathrm{M} \rangle} \,, \qquad [46]$$

$$C_\mathrm{Tol,M} = \frac{\langle \rho_\mathrm{Tol} \rho_\mathrm{M} \rangle - \langle \rho_\mathrm{Tol} \rangle \langle \rho_\mathrm{M} \rangle}{\langle \rho_\mathrm{Tol} \rangle \langle \rho_\mathrm{M} \rangle} \,. \qquad [47]$$

C. Effective recognition rate. In Eq. (6) of the main text, we defined the effective recognition rate as

$$k_\mathrm{rec} = \frac{\langle k_\mathrm{r} \rho_\mathrm{T} \rho_\mathrm{M} \rangle}{\langle \rho_\mathrm{T} \rangle} \,. \qquad [48]$$

We obtain this rate in simulations using the spatial average Eq. Eq. (42). We also note that for the uniform, non-segregated system, the effective recognition rate becomes

$$k_\mathrm{rec}^\mathrm{uniform} = k_\mathrm{r} \rho_\mathrm{M} \,. \qquad [49]$$

5. Thymocyte localization and chemotaxis in the medullary pattern

In this section, we present additional results on self-reactive and self-tolerant thymocyte localization, as well as chemotaxis strength in dependence on the medulla-cortex pattern length scale.

A. Intramedullary thymocyte localization. As outlined in the main text, Sec. III B, chemotaxis drives self–reactive thymocytes to aggregate inside the medulla. In Fig. 4(D), (E) of the main text, we show the average thymocyte densities in the cortex and medulla. However, the two thymocyte species show subtle differences in their distributions within the medulla, as shown in Fig. S6. The concentration of self–reactive thymocytes is highest at the edges of the medulla, and falls off toward the middle, as they are degraded inside the medulla. On the other hand, self–tolerant thymocytes are distributed roughly uniformly throughout the medulla because they are not negatively selected and degraded, and the rate of export into the body is much lower than the degradation rate of self-reactive thymocytes. Should this result hold in the experimental setting, we expect that it has downstream effects on the intra-medullary architecture, for example on distributions of medullary cells that rely on signals from activated thymocytes such as Aire$^+$ mTECs (33, 48).

B. Dependence of chemotaxis on the medullary pattern length scale. As discussed in Sec. III D of the main text, the length scale of the cortex-medulla pattern impacts the effective recognition rate of self–reactive thymocytes. We relate the increase in the effective recognition rate to a faster transition of the thymocytes from the cortex into the medulla. Here, we measure the average chemotactic speed $\langle |\boldsymbol{v}_\mathrm{C}| \rangle = \langle T\chi(\rho_\mathrm{C}) \, |\nabla \rho_\mathrm{C}| \rangle$ of the thymocytes in the simulations. Figure S7 shows that the chemotaxis speed is strongly dependent on the length scale of the cortex-medulla pattern. It is small for small pattern length scales (around 200µm), then increases toward intermediate length scales, before falling off at length scale $\gtrsim$ 1mm. The fall-off in the chemotaxis speed at large pattern length scales coincides with the fall-off in the effective recognition rate [cf. Fig. 6(C) in the main text]. The chemotaxis speed also falls off toward short pattern length scales while the effective recognition rate levels off towards the value of homogeneously distributed mTECs for short length–scales (Fig. 6(C), main text). The reason for this difference at short length scales is that chemotaxis becomes less important for the recognition process as the thymocyte densities are smeared out by diffusion.

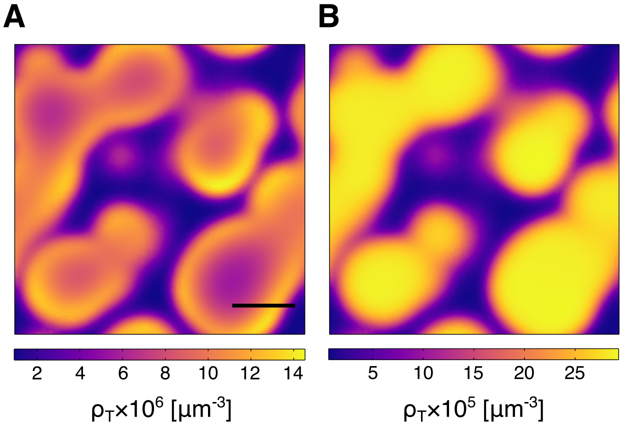

Fig. S6. Intra-medullary localization of self-reactive and tolerant thymocytes. (*A*) Self-reactive thymocyte densities are highest at the medulla edge (cortico-medullary junction, CMJ). This is due to thymocyte flow into the medulla from the edge and degradation in the interior. (*B*) Self-tolerant thymocytes reach the highest density in the interior of the medulla, as their time scale of degradation due to thymus exit is far slower than the time it takes to migrate into the medulla (see also Sec. B.17). C.f. also Fig. 4(*C*), main text. Scale bar $500\mu\mathrm{m}$.

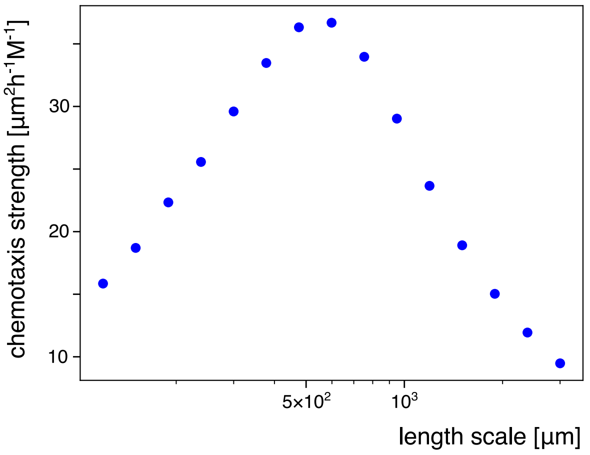

Fig. S7. Medullary length scale modulates chemotaxis speed. The averaged chemotaxis speed $\langle |\boldsymbol{v}_\mathrm{C}| \rangle = \langle T\chi(\rho_\mathrm{C})\, |\nabla \rho_\mathrm{C}| \rangle$ is largest for intermediate length scales of medullary lamellae. The chemotaxis speed peaks at the same medullary length scale [c.f. Fig. 6(*C*), main text] as selection efficiency.

Table S1. Parameters for wt (third column; cf. Fig. 3(*A*) in the main text), and for Fig. 3, and Fig. 4 from the main text. For Figs. 3, 4, the parameters varying from the wt values are shown. Parameters that are not part of the specific model variant are indicated with a dash.

parameter name	symbol	wt values	Fig. 3				Fig. 4		unit
			(*A*)	(*B*)	(*C*)	(*D*)	with chemotaxis	without chemotaxis	
chemokine diffusivity	D_{C}	23400							$\mu\mathrm{m}^2\,\mathrm{h}^{-1}$
thymocyte random motility	D_{T}	24000							$\mu\mathrm{m}^2\,\mathrm{h}^{-1}$
taxis strength	T	10^5						0	$\mu\mathrm{m}^2\,\mathrm{h}^{-1}$
mTEC random motility	D_{M}	3							$\mu\mathrm{m}^2\,\mathrm{h}^{-1}$
receptor binding constant	K_{OffOn}	5							nM
thymocyte production	p	$2.5\cdot10^{-6}$		$2.5\cdot10^{-5}$	10^{-6}	$7\cdot10^{-6}$			$\mu\mathrm{m}^{-3}\,\mathrm{h}^{-1}$
mTEC density scale	μ	10^{-4}							$\mu\mathrm{m}^{-3}$
thymocyte deletion	d	1		10					h^{-1}
thymocyte conversion	k_{r}	$1.5\cdot10^{3}$							$\mu\mathrm{m}^3\,\mathrm{h}^{-1}$
mTEC birth rate	k_{b}	$3\cdot10^{3}$							$\mu\mathrm{m}^3\,\mathrm{h}^{-1}$
mTEC carrying capacity	K	$7.5\cdot10^{-4}$							$\mu\mathrm{m}^{-3}$
mTEC decay	k_{m}	0.005							h^{-1}
chemokine emission	k_{e}	$1.4\cdot10^{-2}$							$\mathrm{M}\,\mu\mathrm{m}^3\,\mathrm{h}^{-1}$
chemokine decay	k_{c}	10							h^{-1}
tolerant thymocyte production	p_{T}^{0}	-	-	-	-	-	$2.5\cdot10^{-6}$	$2.5\cdot10^{-6}$	$\mu\mathrm{m}^{-3}\,\mathrm{h}^{-1}$
thymocyte exit	k_{ex}	-	-	-	-	-	$1/5$	$1/5$	d^{-1}
system side length	L	3							mm
simulated time	$t_{\max}$	2000							h

Table S2. Parameters for wt (third column; cf. Fig. 3(*A*) in the main text), and for Fig. 5, and Fig. 6 from the main text. Parameters varying from the wt values are shown. Parameters that are not part of the specific model variant are indicated with a dash.

parameter name	symbol	wt values	Fig. 5	Fig. 6		unit
				sphere – (*A*, *B*, *D*, *E*)	lamellae – (*C*)	
chemokine diffusivity	D_{C}	23400				$\mathrm{\mu m^2\,h^{-1}}$
thymocyte random motility	D_{T}	24000				$\mathrm{\mu m^2\,h^{-1}}$
taxis strength	T	10^5				$\mathrm{\mu m^2\,h^{-1}}$
mTEC random motility	D_{M}	3				$\mathrm{\mu m^2\,h^{-1}}$
receptor binding constant	K_{OffOn}	5				nM
thymocyte production	p	$2.5\cdot10^{-6}$	$2.5\cdot10^{-7}-2.5\cdot10^{-6}$			$\mathrm{\mu m^{-3}\,h^{-1}}$
mTEC density scale	μ	10^{-4}				$\mathrm{\mu m^{-3}}$
thymocyte deletion	d	1	$0.1-10$			$\mathrm{h^{-1}}$
thymocyte conversion	k_{r}	$1.5\cdot10^3$				$\mathrm{\mu m^3\,h^{-1}}$
mTEC birth rate	k_{b}	$3\cdot10^3$				$\mathrm{\mu m^3\,h^{-1}}$
mTEC carrying capacity	K	$7.5\cdot10^{-4}$				$\mathrm{\mu m^{-3}}$
mTEC decay	k_{m}	0.005				$\mathrm{h^{-1}}$
chemokine emission	k_{e}	$1.4\cdot10^{-2}$				$\mathrm{M\,\mu m^3\,h^{-1}}$
chemokine decay	k_{c}	10				$\mathrm{h^{-1}}$
pattern length scale	L_{p}	-	-	-	$0.12-3$	mm
equilibration time	T_{eq}	-	-	3000	-	h
system side length	L	3	4		1	mm
simulated time	$t_{\max}$	2000		10^4		h

References

1. A Stevens, HG Othmer, Aggregation, Blowup, and Collapse: The ABC's of Taxis in Reinforced Random Walks. *SIAM J. on Appl. Math.* **57**, 1044–1081 (1997).
2. Y Takahama, Journey through the thymus: Stromal guides for T-cell development and selection. *Nat. Rev. Immunol.* **6**, 127–135 (2006).
3. JN Lancaster, Y Li, LIR Ehrlich, Chemokine-Mediated Choreography of Thymocyte Development and Selection. *Trends Immunol.* **39**, 86–98 (2018).
4. S Tanabe, et al., Identification of a new mouse beta-chemokine, thymus-derived chemotactic agent 4, with activity on T lymphocytes and mesangial cells. *The J. Immunol.* **159**, 5671–5679 (1997).
5. T Ueno, et al., Role for CCR7 Ligands in the Emigration of Newly Generated T Lymphocytes from the Neonatal Thymus. *Immunity* **16**, 205–218 (2002).
6. HM Thyagarajan, JN Lancaster, SA Lira, LIR Ehrlich, CCR8 is expressed by post-positive selection CD4-lineage thymocytes but is dispensable for central tolerance induction. *PLOS ONE* **13**, e0200765 (2018).
7. S Ki, HM Thyagarajan, Z Hu, JN Lancaster, LI Ehrlich, EBI2 contributes to the induction of thymic central tolerance in mice by promoting rapid motility of medullary thymocytes. *Eur. J. Immunol.* **47**, 1906–1917 (2017).
8. JE Cowan, et al., Differential Requirement for CCR4 and CCR7 during the Development of Innate and Adaptive $\alpha\beta$T Cells in the Adult Thymus. *The J. Immunol.* **193**, 1204–1212 (2014).
9. Z Hu, JN Lancaster, C Sasiponganan, LI Ehrlich, CCR4 promotes medullary entry and thymocyte–dendritic cell interactions required for central tolerance. *J. Exp. Medicine* **212**, 1947–1965 (2015).
10. JE Park, et al., A cell atlas of human thymic development defines T cell repertoire formation. *Science* **367**, eaay3224 (2020).
11. T Ueno, et al., CCR7 Signals Are Essential for Cortex–Medulla Migration of Developing Thymocytes. *J. Exp. Medicine* **200**, 493–505 (2004).
12. T Nitta, S Nitta, Y Lei, M Lipp, Y Takahama, CCR7-mediated migration of developing thymocytes to the medulla is essential for negative selection to tissue-restricted antigens. *Proc. Natl. Acad. Sci.* **106**, 17129–17133 (2009).
13. LIR Ehrlich, DY Oh, IL Weissman, RS Lewis, Differential Contribution of Chemotaxis and Substrate Restriction to Segregation of Immature and Mature Thymocytes. *Immunity* **31**, 986–998 (2009).
14. J Halkias, et al., Opposing chemokine gradients control human thymocyte migration in situ. *The J. Clin. Investig.* **123**, 2131–2142 (2013).
15. Y Li, et al., CCR4 and CCR7 differentially regulate thymocyte localization with distinct outcomes for central tolerance. *eLife* **12**, e80443 (2023).
16. A Misslitz, et al., Thymic T Cell Development and Progenitor Localization Depend on CCR7. *J. Exp. Medicine* **200**, 481–491 (2004).
17. M Kozai, et al., Essential role of CCL21 in establishment of central self-tolerance in T cells. *J. Exp. Medicine* **214**, 1925–1935 (2017).
18. N Lopes, J Charaix, O Cédile, A Sergé, M Irla, Lymphotoxin α fine-tunes T cell clonal deletion by regulating thymic entry of antigen-presenting cells. *Nat. Commun.* **9**, 1262 (2018).
19. T Baba, Y Nakamoto, N Mukaida, Crucial Contribution of Thymic Sirpα+ Conventional Dendritic Cells to Central Tolerance against Blood-Borne Antigens in a CCR2-Dependent Manner. *The J. Immunol.* **183**, 3053–3063 (2009).
20. O Cédile, LØ Jørgensen, I Frank, A Wlodarczyk, T Owens, The chemokine receptor CCR2 maintains plasmacytoid dendritic cell homeostasis. *Immunol. Lett.* **192**, 72–78 (2017).
21. O Cédile, et al., CCL2 chemokine in T cell tolerance and protection against experimental autoimmune encephalomyelitis. *J. Neuroimmunol.* **275**, 197 (2014).
22. TM McCaughtry, TA Baldwin, MS Wilken, KA Hogquist, Clonal deletion of thymocytes can occur in the cortex with no involvement of the medulla. *J. Exp. Medicine* **205**, 2575–2584 (2008).
23. C Ardavi´n, Thymic dendritic cells. *Immunol. Today* **18**, 350–361 (1997).
24. Y Takahama, I Ohigashi, S Baik, G Anderson, Generation of diversity in thymic epithelial cells. *Nat. Rev. Immunol.* **17**, 295–305 (2017).
25. N Kadouri, S Nevo, Y Goldfarb, J Abramson, Thymic epithelial cell heterogeneity: TEC by TEC. *Nat. Rev. Immunol.* **20**, 239–253 (2020).
26. P Brennecke, et al., Single-cell transcriptome analysis reveals coordinated ectopic gene-expression patterns in medullary thymic epithelial cells. *Nat. Immunol.* **16**, 933–941 (2015).
27. C Bornstein, et al., Single-cell mapping of the thymic stroma identifies IL-25-producing tuft epithelial cells. *Nature* **559**, 622–626 (2018).
28. J Baran-Gale, et al., Ageing compromises mouse thymus function and remodels epithelial cell differentiation. *eLife* **9**, e56221 (2020).
29. KL Wells, et al., Combined transient ablation and single-cell RNA-sequencing reveals the development of medullary thymic epithelial cells. *eLife* **9**, e60188 (2020).
30. EW Shores, W Van Ewijk, A Singer, Disorganization and restoration of thymic medullary epithelial cells in T cell receptor-negative scid mice: Evidence that receptor-bearing lymphocytes influence maturation of the thymic microenvironment. *Eur. J. Immunol.* **21**, 1657–1661 (1991).

31. M Irla, et al., Autoantigen-Specific Interactions with CD4+ Thymocytes Control Mature Medullary Thymic Epithelial Cell Cellularity. *Immunity* **29**, 451–463 (2008).
32. M Irla, et al., Antigen Recognition By Autoreactive Cd4+ Thymocytes Drives Homeostasis Of The Thymic Medulla. *PLoS ONE* **7**, e52591 (2012).
33. N Lopes, et al., Thymocytes trigger self-antigen-controlling pathways in immature medullary thymic epithelial stages. *eLife* **11**, e69982 (2022).
34. L Klein, B Kyewski, PM Allen, KA Hogquist, Positive and negative selection of the T cell repertoire: What thymocytes see (and don't see). *Nat. Rev. Immunol.* **14**, 377–391 (2014).
35. MS Anderson, et al., Projection of an Immunological Self Shadow Within the Thymus by the Aire Protein. *Science* **298**, 1395–1401 (2002).
36. L Onder, et al., Alternative NF-κB signaling regulates mTEC differentiation from podoplanin-expressing precursors in the cortico-medullary junction. *Eur. J. Immunol.* **45**, 2218–2231 (2015).
37. G Anderson, Y Takahama, Thymic epithelial cells: Working class heroes for T cell development and repertoire selection. *Trends Immunol.* **33**, 256–263 (2012).
38. H Takaba, et al., Fezf2 Orchestrates a Thymic Program of Self-Antigen Expression for Immune Tolerance. *Cell* **163**, 975–987 (2015).
39. KM Ashby, KA Hogquist, A guide to thymic selection of T cells. *Nat. Rev. Immunol.* pp. 1–15 (2023).
40. ER Breed, M Watanabe, KA Hogquist, Measuring Thymic Clonal Deletion at the Population Level. *The J. Immunol.* **202**, 3226–3233 (2019).
41. JN Lancaster, et al., Live-cell imaging reveals the relative contributions of antigen-presenting cell subsets to thymic central tolerance. *Nat. Commun.* **10**, 2220 (2019).
42. JP van Meerwijk, et al., Quantitative Impact of Thymic Clonal Deletion on the T Cell Repertoire. *J. Exp. Medicine* **185**, 377–384 (1997).
43. M Hinterberger, et al., Autonomous role of medullary thymic epithelial cells in central CD4+ T cell tolerance. *Nat. Immunol.* **11**, 512–519 (2010).
44. JS Perry, et al., Distinct Contributions of Aire and Antigen-Presenting-Cell Subsets to the Generation of Self-Tolerance in the Thymus. *Immunity* **41**, 414–426 (2014).
45. H Wang, JC Zúñiga-Pflücker, Thymic Microenvironment: Interactions Between Innate Immune Cells and Developing Thymocytes. *Front. Immunol.* **13** (2022).
46. N Lopes, A Sergé, P Ferrier, M Irla, Thymic Crosstalk Coordinates Medulla Organization and T-Cell Tolerance Induction. *Front. Immunol.* **6** (2015).
47. D Malhotra, et al., Tolerance is established in polyclonal CD4+ T cells by distinct mechanisms, according to self-peptide expression patterns. *Nat. Immunol.* **17**, 187–195 (2016).
48. M Irla, et al., Three-Dimensional Visualization of the Mouse Thymus Organization in Health and Immunodeficiency. *The J. Immunol.* **190**, 586–596 (2013).
49. M Sakata, I Ohigashi, Y Takahama, Cellularity of Thymic Epithelial Cells in the Postnatal Mouse. *The J. Immunol.* **200**, 1382–1388 (2018).
50. JA Williams, et al., Thymic Medullary Epithelium and Thymocyte Self-Tolerance Require Cooperation between CD28–CD80/86 and CD40–CD40L Costimulatory Pathways. *The J. Immunol.* **192**, 630–640 (2014).
51. SR Daley, DY Hu, CC Goodnow, Helios marks strongly autoreactive CD4+ T cells in two major waves of thymic deletion distinguished by induction of PD-1 or NF-κB. *J. Exp. Medicine* **210**, 269–285 (2013).
52. GL Stritesky, et al., Murine thymic selection quantified using a unique method to capture deleted T cells. *Proc. Natl. Acad. Sci.* **110**, 4679–4684 (2013).
53. IL Dzhagalov, KG Chen, P Herzmark, EA Robey, Elimination of Self-Reactive T Cells in the Thymus: A Timeline for Negative Selection. *PLoS Biol.* **11**, e1001566 (2013).
54. NS Kurd, et al., A role for phagocytosis in inducing cell death during thymocyte negative selection. *eLife* **8**, e48097 (2019).
55. MÈ Lebel, et al., Differential expression of tissue-restricted antigens among mTEC is associated with distinct autoreactive T cell fates. *Nat. Commun.* **11**, 3734 (2020).
56. JL Bautista, et al., Intraclonal competition limits the fate determination of regulatory T cells in the thymus. *Nat. Immunol.* **10**, 610–617 (2009).
57. JJ Moon, et al., Quantitative impact of thymic selection on Foxp3+ and Foxp3- subsets of self-peptide/MHC class II-specific CD4+ T cells. *Proc. Natl. Acad. Sci.* **108**, 14602–14607 (2011).
58. FP Legoux, et al., CD4 + T Cell Tolerance to Tissue-Restricted Self Antigens Is Mediated by Antigen-Specific Regulatory T Cells Rather Than Deletion. *Immunity* **43**, 896–908 (2015).
59. TM McCaughtry, MS Wilken, KA Hogquist, Thymic emigration revisited. *J. Exp. Medicine* **204**, 2513–2520 (2007).
60. J DeKoning, et al., Thymic cortical epithelium is sufficient for the development of mature T cells in relB-deficient mice. *The J. Immunol.* **158**, 2558–2566 (1997).
61. DHD Gray, et al., Developmental kinetics, turnover, and stimulatory capacity of thymic epithelial cells. *Blood* **108**, 3777–3785 (2006).
62. D Gray, J Abramson, C Benoist, D Mathis, Proliferative arrest and rapid turnover of thymic epithelial cells expressing Aire. *J. Exp. Medicine* **204**, 2521–2528 (2007).

63. M Dumont-Lagacé, S Brochu, C St-Pierre, C Perreault, Adult Thymic Epithelium Contains Nonsenescent Label-Retaining Cells. *The J. Immunol.* **192**, 2219–2226 (2014).
64. AM Gallegos, MJ Bevan, Central Tolerance to Tissue-specific Antigens Mediated by Direct and Indirect Antigen Presentation. *J. Exp. Medicine* **200**, 1039–1049 (2004).
65. T Venables, AV Griffith, A DeAraujo, HT Petrie, Dynamic changes in epithelial cell morphology control thymic organ size during atrophy and regeneration. *Nat. Commun.* **10**, 4402 (2019).
66. M Hirakawa, et al., Fundamental parameters of the developing thymic epithelium in the mouse. *Sci. Reports* **8**, 11095 (2018).
67. M Anderson, SK Anderson, AG Farr, Thymic vasculature: Organizer of the medullary epithelial compartment? *Int. Immunol.* **12**, 1105–1110 (2000).
68. Y Wang, DJ Irvine, Convolution of chemoattractant secretion rate, source density, and receptor desensitization direct diverse migration patterns in leukocytes. *Integr. Biol.* **5**, 481–494 (2013).
69. AM Lambeir, et al., Kinetic Investigation of Chemokine Truncation by CD26/Dipeptidyl Peptidase IV Reveals a Striking Selectivity within the Chemokine Family *. *J. Biol. Chem.* **276**, 29839–29845 (2001).
70. BA Zabel, et al., Chemoattractants, extracellular proteases, and the integrated host defense response. *Exp. Hematol.* **34**, 1021–1032 (2006).
71. I Comerford, S Milasta, V Morrow, G Milligan, R Nibbs, The chemokine receptor CCX-CKR mediates effective scavenging of CCL19 in vitro. *Eur. J. Immunol.* **36**, 1904–1916 (2006).
72. MD Bunting, et al., CCX-CKR deficiency alters thymic stroma impairing thymocyte development and promoting autoimmunity. *Blood* **121**, 118–128 (2013).
73. B Lucas, et al., CCRL1/ACKR4 is expressed in key thymic microenvironments but is dispensable for T lymphopoiesis at steady state in adult mice. *Eur. J. Immunol.* **45**, 574–583 (2015).
74. uniprot, Ccl21a - C-C motif chemokine 21a - Mus musculus (Mouse) | UniProtKB | UniProt (https://www.uniprot.org/uniprotkb/P84444/entry) (2023).
75. X Zhao, S Jain, H Benjaminlarman, S Gonzalez, D Irvine, Directed cell migration via chemoattractants released from degradable microspheres. *Biomaterials* **26**, 5048–5063 (2005).
76. DA Lauffenburger, RT Tranquillo, SH Zigmond, [9] Concentration gradients of chemotactic factors in chemotaxis assays in *Methods in Enzymology*, Immunochemical Techniques Part L: Chemotaxis and Inflammation. (Academic Press) Vol. 162, pp. 85–101 (1988).
77. C Tanford, ML Huggins, Physical Chemistry of Macromolecules. *J. The Electrochem. Soc.* **109**, 98C (1962).
78. M Le Borgne, et al., The impact of negative selection on thymocyte migration in the medulla. *Nat. Immunol.* **10**, 823–830 (2009).
79. K Willimann, et al., The chemokine SLC is expressed in T cell areas of lymph nodes and mucosal lymphoid tissues and attracts activated T cells via CCR7. *Eur. J. Immunol.* **28**, 2025–2034 (1998).
80. TR Ott, et al., The N-terminal domain of CCL21 reconstitutes high affinity binding, G protein activation, and chemotactic activity, to the C-terminal domain of CCL19. *Biochem. Biophys. Res. Commun.* **348**, 1089–1093 (2006).
81. R Yoshida, et al., Secondary Lymphoid-tissue Chemokine Is a Functional Ligand for the CC Chemokine Receptor CCR7 *. *J. Biol. Chem.* **273**, 7118–7122 (1998).
82. U Haessler, M Pisano, M Wu, MA Swartz, Dendritic cell chemotaxis in 3D under defined chemokine gradients reveals differential response to ligands CCL21 and CCL19. *Proc. Natl. Acad. Sci.* **108**, 5614–5619 (2011).
83. LA Segel, A Theoretical Study of Receptor Mechanisms in Bacterial Chemotaxis. *SIAM J. on Appl. Math.* **32**, 653–665 (1977).
84. MC Cross, PC Hohenberg, Pattern formation outside of equilibrium. *Rev. Mod. Phys.* **65**, 851–1112 (1993).
85. AM Turing, The chemical basis of morphogenesis. *Philos. Transactions Royal Soc. London. Ser. B, Biol. Sci.* **237**, 37–72 (1952).
86. LA Segel, JL Jackson, Dissipative structure: An explanation and an ecological example. *J. Theor. Biol.* **37**, 545–559 (1972).
87. A Gierer, H Meinhardt, A theory of biological pattern formation. *Kybernetik* **12**, 30–39 (1972).
88. JD Murray, ed., *Mathematical Biology: II: Spatial Models and Biomedical Applications*, Interdisciplinary Applied Mathematics. (Springer, New York, NY) Vol. 18, (2003).
89. EF Keller, LA Segel, Initiation of slime mold aggregation viewed as an instability. *J. Theor. Biol.* **26**, 399–415 (1970).
90. EF Keller, LA Segel, Model for chemotaxis. *J. Theor. Biol.* **30**, 225–234 (1971).
91. T Hillen, KJ Painter, A user's guide to PDE models for chemotaxis. *J. Math. Biol.* **58**, 183–217 (2009).
92. H Weyer, D Muramatsu, E Frey, Chemotaxis-induced phase separation (2024).
93. H Weyer, D Muramatsu, E Frey, Coarsening dynamics of chemotactic aggregates (2024).
94. COMSOL Multiphysics (COMSOL AB) (2021).
95. HR Rodewald, S Paul, C Haller, H Bluethmann, C Blum, Thymus medulla consisting of epithelial islets each derived from a single progenitor. *Nature* **414**, 763–768 (2001).
